# Advancements in polymer-based drug delivery systems targeting the tumor microenvironment for immunotherapy of triple-negative breast cancer

**DOI:** 10.3389/fimmu.2026.1927103

**Published:** 2026-09-23

**Authors:** Zhenxiao Wang, Mo Li, Zhenyu Wang, Ying Wang

**Affiliations:** 1 Department of Hepatobiliary and Pancreatic Surgery, The Second Hospital of Jilin University, Changchun, Jilin, China; 2 Department of Thyroid Surgery, The Second Hospital of Jilin University, Changchun, Jilin, China; 3 Department of Breast Surgery, The Second Hospital of Jilin University, Changchun, Jilin, China

**Keywords:** immunosuppression, immunotherapy, metabolic reprogramming, nanomedicine, polymer-based drug delivery systems, triple-negative breast cancer, tumor microenvironment

## Abstract

Triple-negative breast cancer (TNBC) is an aggressive breast cancer subtype characterized by high invasiveness, frequent recurrence, and metastasis, along with limited targeted therapeutic options. Despite clinical advances achieved with immune checkpoint inhibitors, poly(ADP-ribose) polymerase (PARP) inhibitors, and antibody-drug conjugates, the complex immunosuppressive tumor microenvironment (TME) of TNBC hinders drug delivery and compromises the efficacy of immunotherapy. Multiple adverse features of the TME, including impaired tumor immunogenicity, dysfunctional antigen presentation, dense extracellular matrix barriers, abundant immunosuppressive cell populations, metabolic reprogramming, hypoxia, and acidification, collectively contribute to immunotherapy resistance. Polymer-based drug delivery systems (PBDS), with tunable physicochemical properties, structural versatility, high drug-loading capacity, and responsiveness to TME-associated stimuli, have emerged as promising platforms for remodeling the TNBC TME. These polymeric carriers can achieve tumor enrichment, enable controlled release, improve deep tissue penetration, and facilitate co-delivery of multiple therapeutic agents to exert synergistic effects. In this review, we adopt a barrier-to-design framework that directly links the major immunosuppressive barriers of the TNBC TME to corresponding polymer-engineering strategies. Unlike carrier-centered reviews that primarily catalog nanomaterial classes, we critically compare stimuli-responsive activation, sequential release, biomimetic targeting, local hydrogel depots, spatial targeting and multifunctional co-delivery according to their mechanistic rationale, preclinical evidence, model dependence, safety and translational feasibility. We further discuss subtype- and biomarker-informed patient selection, design requirements for primary tumors, tumor-draining lymph nodes and metastatic niches, and the need to balance multifunctionality against manufacturability. This framework provides a clinically oriented roadmap for developing barrier-matched PBDS as rational partners for TNBC immunotherapy.

## Graphical abstract

**Figure:**
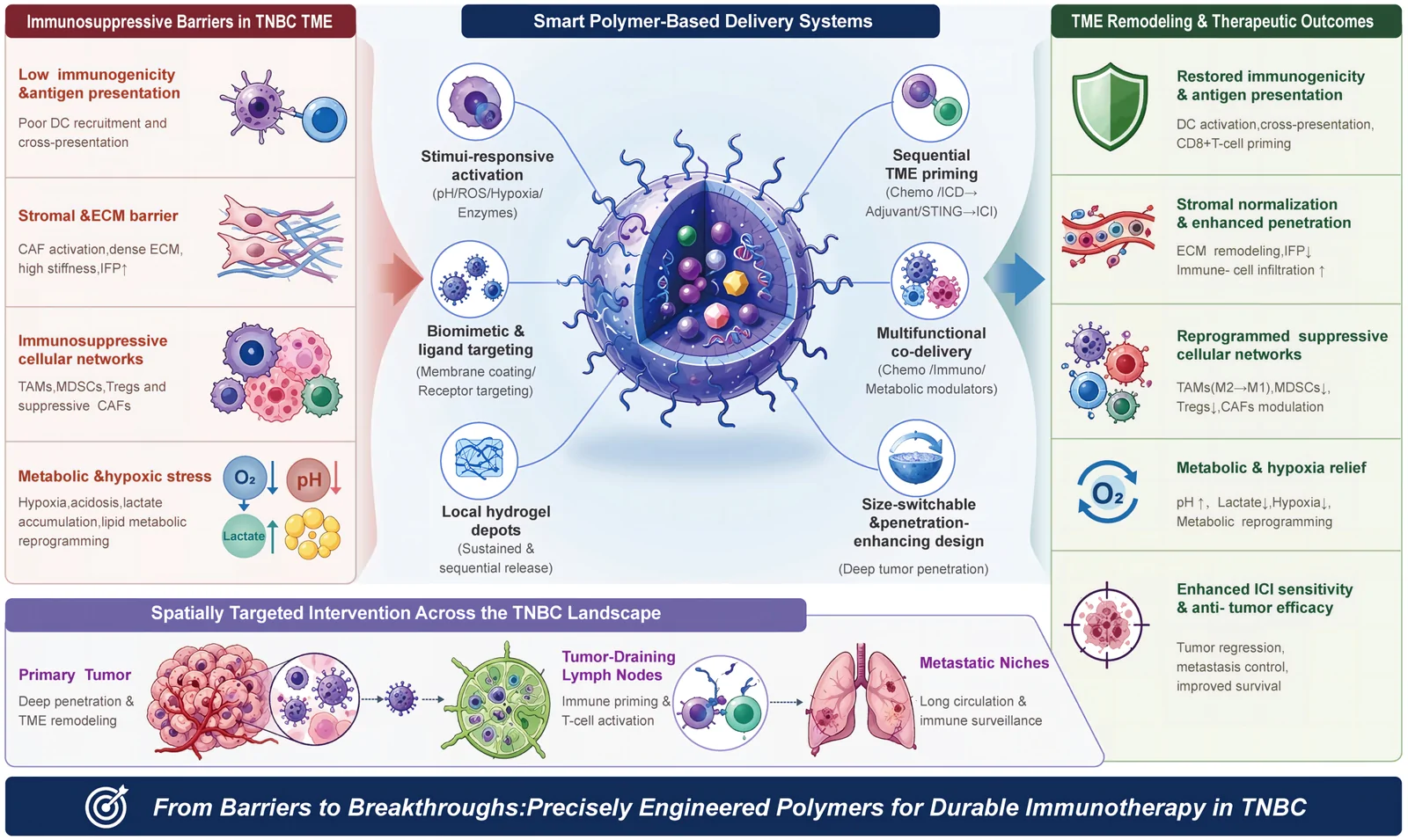
Barrier-matched polymer engineering for TNBC immunotherapy. Major immune, stromal and metabolic barriers in the TNBC TME are matched to modular PBDS strategies—including smart polymer nanoparticles, biomimetic coatings, local hydrogels and stimuli-responsive release—to drive TME remodeling, enhance checkpoint-blockade sensitivity and reduce recurrence/metastasis. Biomarker selection, spatial targeting, biosafety and manufacturability act as translational filters.

## 1 Introduction

Triple-negative breast cancer (TNBC) represents one of the most aggressive and heterogeneous molecular subtypes of breast cancer. It is defined by the absence of expression of the estrogen receptor, progesterone receptor, and human epidermal growth factor receptor 2 (HER2) and accounts for approximately 15%–20% of all breast cancer cases (1). Compared to hormone receptor-positive (HR^+^) or HER2-positive breast cancer, TNBC is more prevalent among younger patients. Moreover, it is associated with clinical features such as high histological grade, active proliferation, early recurrence, and an elevated risk of visceral and brain metastases (2). Recent advances in understanding TNBC molecular typing, immune microenvironment characteristics, and drug-targetable pathways have transformed TNBC from a “target-free” disease into a complex disease spectrum. These include strategies targeting immune pathways, DNA damage repair mechanisms, trophoblast cell-surface antigen 2 (TROP2), epidermal growth factor receptor (EGFR), and metabolic pathways (3, 4).

Significant progress has recently been achieved in TNBC therapy. Integrating immune checkpoint inhibitors (ICIs), such as pembrolizumab, with chemotherapy has emerged as a pivotal treatment strategy for patients with high-risk early-stage TNBC and PD-L1-positive metastatic TNBC (5). Additionally, PARP inhibitors are approved for patients with HER2-negative breast cancer who possess germline breast cancer susceptibility gene 1/2 (BRCA1/2) mutations. Moreover, antibody-drug conjugates (ADCs), including sacituzumab govitecan and certain HER2-low-related ADCs, offer novel therapeutic options for refractory TNBC (6). However, these advances also indicate that TNBC cannot be effectively managed solely through systemic drug delivery or through a single immune checkpoint blockade (ICB).

The primary challenge in treating TNBC lies in its complex and dynamically evolving tumor microenvironment (TME). This environment comprises various components, including tumor cells, tumor-associated macrophages (TAMs), myeloid-derived suppressor cells (MDSCs), dendritic cells (DCs), regulatory T cells (Tregs), cancer-associated fibroblasts (CAFs), vascular cells, the extracellular matrix (ECM), as well as various cytokines, chemokines, and metabolites (7). Collectively, these components contribute to the limited efficacy of therapeutic interventions such as ICIs, cell therapy, and tumor vaccines by fostering an “immune cold” phenotype. This phenotype is characterized by impaired antigen presentation, depletion of effector T cells, aggregation of immunosuppressive cells, and the establishment of physical and metabolic barriers (8). Although TNBC generally exhibits higher immune infiltration and immunogenicity than certain HR+ breast cancers, many TNBC tumors nevertheless display an inflamed but dysfunctional or immune-excluded phenotype. This phenotype is characterized by PD-L1 upregulation, T-cell dysfunction, myeloid suppression, fibrotic barriers, and metabolic immunosuppression (7, 9, 10). Hypoxia, acidic pH, lactic acid accumulation, and redox imbalance further impair the function of CD8^+^ T cells and natural killer (NK) cells, thereby facilitating tumor cell survival, invasion, and metastasis (11). Furthermore, tumor-draining lymph nodes (TDLNs) and distant pre-metastatic niches can be reprogrammed into immunosuppressive states by TNBC, thereby compromising systemic antitumor immunity (12). Emerging evidence also suggests that the gut microbiome plays a role in estrogen metabolism, regulation of the inflammatory state, immune modulation, and therapeutic response in TNBC (13–15).

To precisely target the TME and address the aforementioned challenges, polymer-based drug delivery systems (PBDS) offer several distinct advantages, including tunable physicochemical properties, controlled release, facile surface modification, and responsiveness to TME stimuli (16). Unlike conventional small-molecule therapeutics that typically target a single pathway, polymeric nanocarriers—including nanoparticles, micelles, hydrogels, and related platforms—enable the co-delivery of chemotherapeutic agents, ICIs, cytokines, and immune adjuvants. Polymeric architectures discussed in this review include nanoparticles, micelles, polymer conjugates, nanogels and injectable/local hydrogels. Importantly, these formats should not be selected as isolated material categories; rather, their physicochemical properties and route of administration should be matched to the dominant biological barrier, desired spatial distribution and release sequence (17). Polymeric carriers may exploit enhanced permeability and retention (EPR)-mediated passive accumulation in some tumors; however, the magnitude and reproducibility of the EPR effect are highly heterogeneous and are generally less predictable in patients than in many preclinical models. Active targeting and stimuli-responsive designs may improve cellular uptake, retention, or site-specific payload release after systemic exposure, but they should be regarded as complementary strategies rather than substitutes for rigorous pharmacokinetic, biodistribution, and safety evaluation (18–21). Recent advancements in nanotechnology-based TNBC immunotherapy approaches have demonstrated their potential to synergistically enhance ICI efficacy, reprogram CAFs, modulate TAM polarization, and remodel the local immune microenvironment. These findings provide a novel strategy for overcoming the limitations of traditional therapies (16).

PBDS designed for the TNBC TME have evolved from systems focused mainly on tumor accumulation to platforms that can also modulate immune, stromal, and metabolic features (22). Important barriers to translation remain, including carrier instability during systemic circulation, protein-corona-mediated clearance, inconsistent performance across preclinical models, TNBC heterogeneity, and the limited availability of models that reproduce human immunity (23, 24). Recent reviews have addressed nanomaterial-based TNBC therapy, the breast cancer TME, or polymeric nanomedicine across tumor types. Here, we instead organize the field around the TME barrier being addressed and examine how polymer design is adapted to that barrier. Representative systems are compared with respect to design, biological target, preclinical limitations, and translational feasibility, with particular attention to their integration with established TNBC therapies.

## 2 Immunosuppressive mechanisms in the TNBC TME

Although these processes are discussed separately for clarity, the TNBC TME is shaped by a coupled network of immunological, stromal and metabolic barriers rather than by isolated defects. Heterogeneous and often insufficient effective immunogenicity limits productive tumor-specific T-cell responses; defective dendritic-cell recruitment and maturation further compromises antigen cross-presentation; CAF-driven ECM remodeling creates a spatial barrier to immune-cell trafficking and drug penetration; TAMs, MDSCs, Tregs and suppressive CAF subsets reinforce inhibitory cytokine and nutrient-depletion circuits; and hypoxia, acidosis, lactate accumulation and lipid-metabolic rewiring progressively impair effector lymphocyte function. These layers interact bidirectionally—for example, hypoxia and lactate promote myeloid suppression, whereas CAF-rich stroma worsens perfusion and metabolic stress—thereby stabilizing immune-excluded and therapy-resistant states. **Figure 1** summarizes these interconnected barriers and provides the biological basis for the barrier-matched polymer design strategies discussed in Section 3.

**Figure 1:**
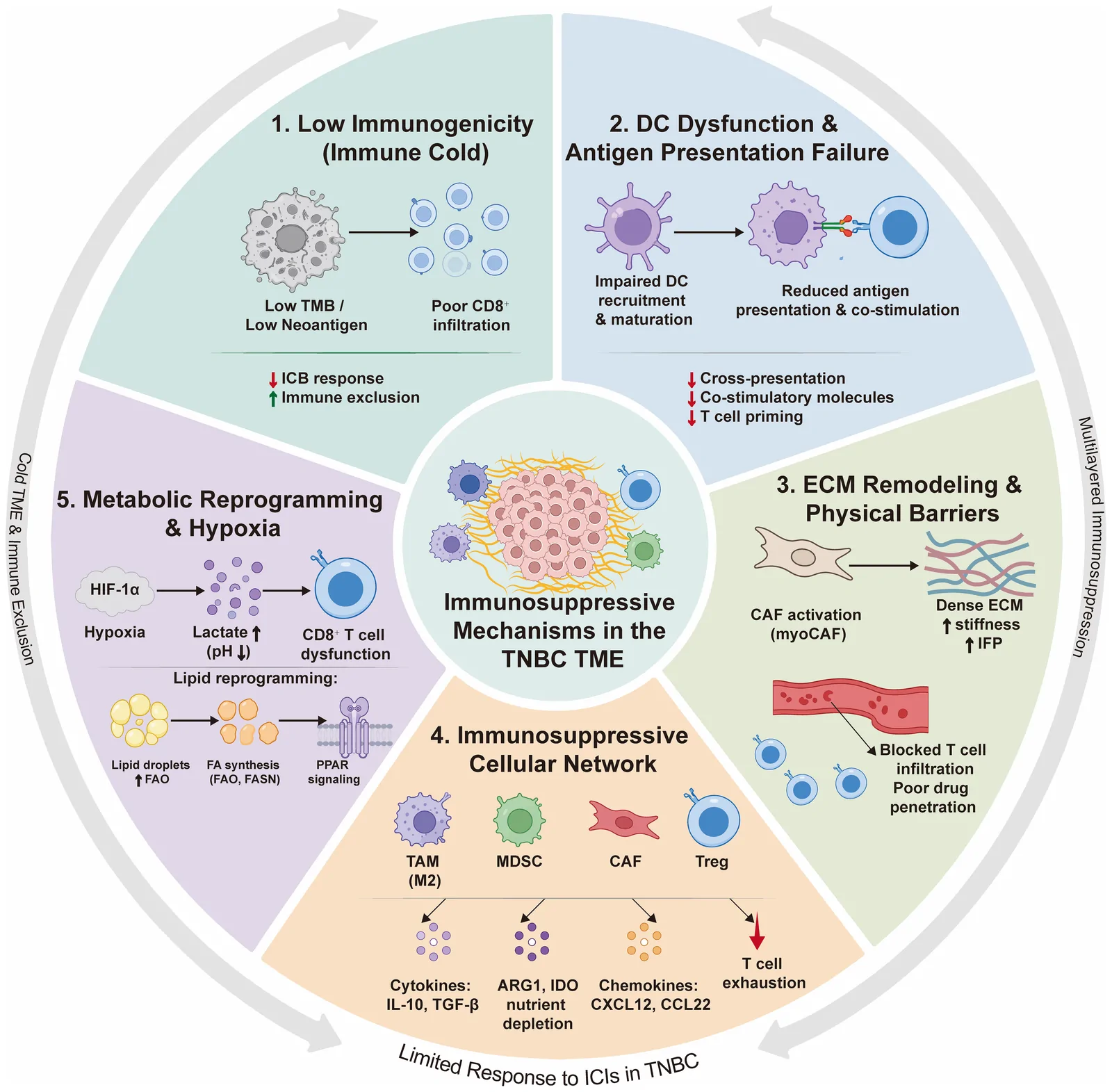
Immunosuppressive mechanisms within the TME of TNBC. The TNBC TME is governed by multiple interconnected immunosuppressive mechanisms that collectively impair antitumor immunity and limit the efficacy of ICIs. Low TMB, limited neoantigen generation, insufficient CD8^+^ T-cell infiltration, and weak immune activation contribute to an “immune-cold” phenotype. DC dysfunction, characterized by impaired recruitment, maturation, antigen cross-presentation, MHC expression, and CD80/CD86-mediated costimulation, further compromises T-cell priming. CAF-driven ECM remodeling increases stromal stiffness and interstitial pressure, thereby restricting CD8^+^ T-cell infiltration and drug penetration. In parallel, TAMs, MDSCs, Tregs, and CAFs establish an immunosuppressive cellular network through IL-10, TGF-β, ROS, nutrient depletion, PD-1/PD-L1 signaling, and myeloid-mediated suppression, ultimately promoting T-cell exhaustion. Metabolic reprogramming, lactate accumulation, acidic pH, lipid metabolic alterations, FAO, hypoxia, and HIF-1α activation further suppress CD8^+^ T-cell and NK-cell function. Together, these mechanisms drive immune exclusion, sustain an immunosuppressive TME, and contribute to ICI resistance in TNBC.

### 2.1 Deficiency in tumor immunogenicity and the ‘immune cold’ phenotype

Although TNBC is often considered one of the more immunogenic subtypes of breast cancer, it is still characterized by overall limited immunogenicity and a weakly inflamed TME, which remain major barriers to effective immunotherapy. In contrast to highly mutated “hot” tumors such as melanoma and non-small cell lung cancer, TNBC exhibits a relatively low tumor mutational burden (TMB) and contains only a limited subset of tumor cell populations capable of generating immunogenic neoantigens that can drive robust adaptive immune responses (25). Multiple studies have failed to demonstrate a consistent correlation between neoantigen load and CD8^+^ T cell infiltration in breast cancer, thereby undermining the neoantigen-driven T cell response hypothesis. A comprehensive pan-cancer analysis by McGrail et al. further supports this observation. In the TMB-high (TMB-H) subgroup of breast cancer, the objective response rate (ORR) to ICB was only 15.3%, even lower than that observed in TMB-low (TMB-L) tumors, and was associated with poorer overall survival (OS). Notably, in the metastatic TNBC subgroup, none of the 10 TMB-H tumors responded to ICB, whereas the ORR in TMB-L tumors reached 20.5% (26). These findings indicate that TMB-H status alone is insufficient to predict effective neoantigen-driven antitumor immunity and represents a key limitation underlying the suboptimal clinical benefit of ICB in TNBC. Supporting this view, Vougiouklakis et al. performed a detailed genomic characterization of 527 TMB-H (≥10 mut/Mb) breast cancer cases. They demonstrated that, although the TNBC subtype exhibited the highest degree of immune cell infiltration among TMB-H tumors, overall TMB in breast cancer remained substantially lower than that observed in melanoma or lung cancer. While apolipoprotein B mRNA editing enzyme, catalytic polypeptide-like mutational signatures predominated in TMB-H tumors, concurrent loss-of-function alterations in AT-rich interaction domain 1A and phosphatase and tensin homolog (PTEN) were frequently associated with immune cell exclusion. Collectively, these genomic features were associated with persistently low ICB response rates (≤20%). This is further supported by clinical evidence, including the KEYNOTE-012 trial, which reported an ORR of only 18.5% with single-agent pembrolizumab in PD-L1-positive metastatic TNBC, as well as the modest survival benefits observed in IMpassion130 and the lack of significant benefit in IMpassion131 (27). Collectively, these data suggest that even in TMB-high TNBC, increased mutational load alone is insufficient to overcome the physical and immunosuppressive barriers within the TME, making TMB an unreliable standalone predictive biomarker for ICB efficacy.

The immunologically “cold” phenotype of TNBC is a multifactorial state driven by coordinated immune dysregulation across the lymph node–tumor axis, fibrotic and immunological exclusion barriers, myeloid-mediated immunosuppression (including TAM and MDSC accumulation), and impaired antigen presentation. Consequently, approaches focused solely on enhancing tumor-cell immunogenicity are generally insufficient to restore effective antitumor immunity (28). Conversely, polymer-based delivery systems capable of precise spatiotemporal intervention at multiple key nodes within the TME offer greater promise for achieving meaningful “cold-to-hot” conversion in TNBC immunotherapy. These platforms can simultaneously induce ICD, enhance DC maturation and antigen presentation, reprogram TAM polarization toward an antitumor phenotype, relieve T-cell exclusion, and potentiate responses to ICB (29, 30). By targeting the interconnected immunosuppressive networks that sustain immune evasion, even in relatively more immunogenic TNBC subtypes, TME-targeted approaches have the potential to overcome the limitations of current ICB-based strategies and improve clinical outcomes in this aggressive breast cancer subtype.

### 2.2 DC dysfunction and antigen presentation failure

DCs are the central antigen-presenting cells that bridge innate and adaptive immunity. Their dysfunction and impaired antigen presentation represent a key mechanism limiting the establishment of effective antitumor immune responses in the TNBC TME (31). Under normal physiological conditions, DCs efficiently take up, process, and present tumor-associated antigens via major histocompatibility complex (MHC) molecules, while simultaneously delivering costimulatory signals and secreting key cytokines, such as interleukin (IL)-12. This coordinated process primes naïve T cells and initiates tumor-specific immune responses (32). In the TNBC TME, however, DCs frequently exhibit impaired recruitment, arrested maturation, reduced cross-presentation capacity, and decreased expression of costimulatory molecules such as CD80 and CD86. These deficiencies disrupt the antitumor immune cycle at its earliest stage. The TNBC TME is enriched with multiple immunosuppressive mediators, including lactate, vascular endothelial growth factor (VEGF), IL-10, transforming growth factor-β (TGF-β), and hypoxia, all of which collectively inhibit DC maturation, antigen processing, and presentation through multiple signaling pathways (25, 33). Consequently, the activation of naïve T cells is either completely suppressed or restricted to low-affinity, ineffective responses.

Compounding these DC-intrinsic impairments, cancer cells in TNBC frequently harbor intrinsic defects in antigen-processing machinery and exhibit downregulated MHC class I expression. These tumor-intrinsic alterations diminish direct antigen presentation to effector CD8^+^ T cells. Furthermore, these alterations reduce the pool of properly processed tumor antigens available for DC-mediated cross-presentation, further compromising efficient priming of cytotoxic CD8^+^ T cells. The synergistic action of DC dysfunction, tumor cell-intrinsic defects in antigen presentation, immunosuppressive cellular networks, ECM barriers, and metabolic reprogramming results in insufficient infiltration and activation of effector T cells. This precludes the formation of an inflamed (immunologically “hot”) TME. Although TNBC generally exhibits higher levels of tumor-infiltrating lymphocytes and PD-L1 expression than the HR^+^/HER2^−^ subtype, a substantial proportion of TNBC tumors remain immunologically cold and develop profound primary resistance to PD-1/PD-L1 immune checkpoint blockade (7). These multilayered impairments in antigen presentation and DC function delineate the molecular underpinnings of immune evasion in TNBC. Additionally, these impairments identify actionable targets for the rational design of PBDS aimed at reprogramming the TME (34). By enabling targeted delivery of DC maturation agonists, immunomodulatory inhibitors of immunosuppressive factors, and antigen-capturing nanocarriers, polymer nanodelivery platforms offer a promising strategy to restore DC antigen-presenting function, reverse TME immunosuppression, and enhance the efficacy of immunotherapy in TNBC (35).

### 2.3 Physical and biological barriers formed by ECM remodeling

CAF-driven ECM remodeling constitutes a major physical barrier in the TNBC TME, restricting immune cell infiltration and deep drug penetration. Particularly, the myoCAF subpopulation, characterized by expression of α-smooth muscle actin (α-SMA) and fibroblast activation protein (FAP), secretes large quantities of collagen, fibronectin, and hyaluronic acid. This leads to ECM densification, elevated tissue stiffness, and increased interstitial fluid pressure, thereby physically impeding the extravasation of CD8^+^ T cells from blood vessels and subsequent infiltration into the tumor core (36). These biomechanical alterations also explain why delivery systems that rely solely on the EPR effect typically achieve only peripheral tumor enrichment, with inadequate penetration into the hypoxic core in TNBC (37).

Recent mechanistic studies have identified a critical “nerve–CAFs–collagen” axis that drives immune exclusion in TNBC. Zhang et al. demonstrated that TNBC cell-secreted nerve growth factor activates sensory neurons to release calcitonin gene-related peptide (CGRP). Subsequently, CGRP engages receptor activity-modifying protein 1 (RAMP1) on CAFs, promoting their conversion to a myoCAF phenotype and driving excessive collagen deposition, thereby generating a dense ECM barrier that excludes T cells. Notably, pharmacological blockade of the CGRP–RAMP1 signaling axis effectively reverses this fibrotic remodeling and markedly potentiates the antitumor efficacy of anti-PD-1 immunotherapy (38). Beyond its physical barrier function, the collagen-rich, fibrotic TME impairs the effector function of infiltrating CD8^+^ T cells through metabolic reprogramming. In their study, Tharp et al. demonstrated in breast cancer models that collagen deposition induces arginine depletion and ornithine accumulation in the local microenvironment, suppressing T-cell antitumor activity and revealing a multilayered immunosuppressive mechanism (39). High-resolution spatial single-cell transcriptomic profiling has delineated the profound spatial heterogeneity and functional specialization of CAFs. Using pan-cancer and TNBC-specific spatial scRNA-seq, Ye et al. established that senescent CAFs secrete distinct ECM components that selectively impair NK-cell cytotoxicity. In contrast, inflammatory CAFs drive tumor invasion while sustaining immunosuppression via cytokines such as IL-6 (40).

Collectively, these findings emphasize the TNBC stromal barrier as a major physical barrier to drug delivery and a key determinant of immunotherapy resistance. Accordingly, next-generation polymer-based nanocarriers are designed to penetrate or enzymatically degrade dense ECM, selectively modulate CAF subpopulations, or respond to matrix cues (FAP-targeted, matrix metalloproteinase (MMP)-responsive, size-switchable, or enzyme-sensitive platforms). These approaches offer a promising strategy to overcome the limitations of conventional EPR-mediated delivery. Such rationally designed systems are expected to enable precise spatiotemporal intervention within the TNBC TME and enhance the efficacy of immunotherapeutic regimens (41).

### 2.4 Recruitment and functional dominance of immunosuppressive cellular components

A growing body of evidence indicates that the recruitment and functional predominance of immunosuppressive cellular components constitute a central mechanism underlying primary or secondary resistance of breast cancer, particularly TNBC, to immunotherapy (42). Within the TME, TAMs, MDSCs, CAFs, and Tregs form an immunosuppressive network in breast cancer. This network restricts CD8^+^ T cell infiltration, induces T cell exhaustion, and impairs antigen presentation, thereby diminishing the efficacy of PD-1/PD-L1 blockade (43, 44). De Visser et al. demonstrated that TAMs contribute directly to CD8^+^ T-cell exhaustion and PD-L1 expression by secreting inhibitory mediators, including IL-10, TGF-β, and ROS. Concurrently, MDSCs exacerbate immunosuppression by depleting essential nutrients, such as arginine and tryptophan, and by reinforcing T-cell dysfunction, together with Tregs (45). Kundu et al. further characterized the immunosuppressive interaction network among CAFs, TAMs, MDSCs, and Tregs in breast cancer. Their study revealed that CAFs recruit and induce CD4^+^CD25^+^ T cells to differentiate into Tregs via C-X-C motif chemokine ligand 12 (CXCL12), while simultaneously promoting the polarization of TAMs toward the M2 phenotype characterized by the secretion of IL-10 and C-C motif chemokine ligand 22 (CCL22), among other factors. This polarization is associated with downregulation of MHC-II and defects in antigen presentation. Furthermore, TAMs and MDSCs suppress the proliferation and effector functions of CD8^+^ T cells through arginase 1-mediated arginine depletion and the upregulation of PD-L1, establishing a positive feedback loop that diminishes the efficacy of PD-1/PD-L1 blockade (8). Clinical evidence further supports these mechanisms. In a study by Wilkerson et al., a single-cell analysis was performed on peripheral immune cells from patients with metastatic TNBC undergoing PD-1/PD-L1 combined chemotherapy. The findings indicated that non-responders exhibited an immunosuppressive CD33^+^ myeloid cell program before treatment. During treatment, MDSC expansion and PD-L1 upregulation within the CD33^+^ human leukocyte antigen-DR-negative (HLA-DR^-^) population were observed. The removal of these CD33^+^ cells partially restored T cell effector function, suggesting that primary and secondary drug resistance may be attributed to pre-existing myeloid suppression. Consequently, the blockade of PD-1/PD-L1 alone is often insufficient to overcome the profound immunosuppression in the TME (46).

The limited effectiveness of ICIs in TNBC therefore reflects a coordinated suppressive network dominated by TAMs and MDSCs, reinforced by CAFs and Tregs, and culminating in impaired CD8^+^ T-cell infiltration and function (47). PBDS designed for this setting should do more than increase intratumoral drug concentration; they should selectively reprogram suppressive myeloid cells, interrupt CAF–myeloid crosstalk, limit Treg expansion, and restore antigen presentation and effector T-cell activity (46, 48–51).

### 2.5 Metabolic reprogramming and hypoxia-induced immunosuppressive environment

Metabolic dysregulation within the TNBC TME represents a major barrier to effective antitumor immunity and successful immunotherapy. Tumor cells exhibit increased glucose uptake and lactate production via the Warburg effect, generating an acidic microenvironment that markedly suppresses CD8^+^ T-cell cytokine secretion, proliferation, and cytotoxicity. Extracellular acidification also activates G protein-coupled receptors, including G protein-coupled receptor 132, driving macrophage polarization toward the pro-tumor M2 phenotype and exacerbating immunosuppression (52). Beyond serving as a metabolic by-product, lactate functions as a signaling metabolite, establishing a detrimental “metabolism-immunity” cycle that promotes T cell exhaustion and M2 polarization through mechanisms such as histone lactylation (53). These mechanisms provide a rationale for developing pH-responsive polymeric carriers that enable preferential drug release under acidic conditions and potentially mitigate metabolically associated immunosuppression in TNBC (54, 55).

Beyond glucose metabolism, lipid metabolic reprogramming plays an equally critical role in TNBC progression and immunotherapy resistance by simultaneously sustaining tumor cell plasticity and sculpting an immunosuppressive TME. Lipidomic analyses in TNBC revealed widespread dysregulation in fatty acid oxidation (FAO), excessive lipid droplet (LD) accumulation, and altered cholesterol metabolism. These alterations provide bioenergetic and biosynthetic support for cancer stem cells (CSCs) maintenance, thereby facilitating epithelial-mesenchymal transition, tumor initiation, and resistance to multiple therapies via peroxisome proliferator-activated receptor (PPAR) signaling and related pathways. Notably, they also directly influence infiltrating immune cells, thereby promoting the establishment of a protumorigenic immune niche (56, 57). In the TNBC TME, upregulated lipogenic enzymes such as fatty acid synthase (FASN) and cluster of differentiation 36 (CD36), together with enhanced FAO, promote the production of immunosuppressive lipid mediators, including prostaglandin E2 and 27-hydroxycholesterol. These mediators polarize TAMs toward the M2 phenotype, recruit MDSCs, and impair the metabolic fitness and cytotoxic capacity of CD8^+^ T cells and NK cells through nutrient competition and direct signaling. Emerging evidence from TNBC models indicates that tumor cells surviving initial therapy accumulate LDs enriched in omega-6 fatty acids (arachidonic acid) (58). These lipid stores can reprogram neutrophils and other myeloid populations toward immunosuppressive phenotypes, thereby contributing to resistance against chemotherapy and ICIs. Pharmacological disruption of LD biogenesis or dietary modulation of omega-6 intake has been demonstrated to alleviate this immunosuppressive state and partially restore tumor sensitivity to anti-PD-1 therapy. Additionally, loss of the epithelial transcription factor ovo-like zinc finger 2 (OVOL2) in TNBC enhances FAO, increasing nicotinamide adenine dinucleotide phosphate (NADPH) production and cellular energy availability, thereby sustaining stemness features such as spheroid formation and tumor-initiating capacity. This lipid-driven stemness program is closely linked to immune escape, as CSCs frequently exhibit diminished immunogenicity and heightened resistance to T cell-mediated cytotoxicity (59). Collectively, these mechanisms position lipid metabolism as a promising therapeutic target for polymer-based nanocarriers designed to simultaneously suppress tumor-intrinsic aggressiveness and extrinsic immunosuppressive barriers in TNBC.

Hypoxia and redox imbalance are fundamental drivers of the metabolic and immunological pathology observed in the TNBC TME. Rapid tumor growth, combined with structurally abnormal and poorly perfused neovasculature, stabilizes hypoxia-inducible factor-1α (HIF-1α) and hypoxia-inducible factor-2α (HIF-2α). These transcription factors orchestrate adaptive programs that promote angiogenesis, metabolic adaptation (including enhanced glycolysis and lipid utilization), invasion, metastasis, and survival under stress (60). In TNBC, hypoxia plays a particularly dominant role in establishing an immunosuppressive ecosystem that contributes to primary and acquired resistance to immunotherapy. HIF-1α upregulates immune checkpoint molecules such as PD-L1 on tumor cells and facilitates the recruitment and activation of MDSCs, TAMs, and Tregs via the secretion of VEGF, TGF-β, and adenosine-generating enzymes (61). Moreover, hypoxia directly induces dysfunction in effector CD8^+^ T and NK cells through an epigenetic reprogramming mechanism. Under hypoxic conditions, HIF-1α recruits histone deacetylase 1 (HDAC1) to effector-gene promoters, particularly those of IFNG and TNF, where pre-existing polycomb repressive complex 2 (PRC2) activity facilitates the replacement of activating H3K27ac with repressive H3K27me3, thereby suppressing immune effector expression, including IFN-γ and TNF, while granzyme B expression is also reduced (62). Consequently, effector lymphocytes exhibit reduced cytokine production, exhaustion-like phenotypes, and failure of anti-PD-1 blockade to restore cytotoxicity, as demonstrated in hypoxic TNBC co-culture systems and *in vivo* syngeneic (4T1) and humanized TNBC xenograft models. Clinical correlations in TNBC cohorts further link elevated hypoxia gene signatures with reduced CD8^+^ T cell infiltration, lower IFN-γ expression, and inferior overall and metastasis-free survival (61). In parallel, the nuclear factor erythroid 2-related factor 2 (NRF2)-mediated antioxidant response promotes tumor cell adaptation to hypoxic and oxidative stress conditions and may intersect with redox-sensitive pathways that further modulate tumor–stromal immune interactions (63). Collectively, these interconnected hypoxia–metabolism–immune networks create a hostile niche for effector immune cells while protecting malignant cells. These mechanistic insights indicate the therapeutic potential of advanced polymer-based nanodelivery systems, including hypoxia-cleavable, ROS-responsive, or multifunctional carriers. Such systems can be engineered to co-deliver metabolic inhibitors, HIF pathway modulators, or immunotherapeutics to reprogram the TME and convert immunologically “cold” TNBC tumors into those responsive to ICIs (64).

## 3 Barrier-to-design strategies of TME-targeted polymer-based delivery systems for TNBC immunotherapy

Conventional free-drug therapies or treatments targeting a single mechanism frequently struggle to concurrently meet the therapeutic requirements of efficient local tumor enrichment, deep tissue penetration, and microenvironment remodeling (65). In this regard, polymeric drug delivery systems targeting TNBC’s unique TME have emerged as a pivotal research direction for TNBC immunotherapy. This is attributed to their distinctive advantages in material design flexibility, functional integration, and delivery control (66).

Recent PBDS studies increasingly combine several engineering principles. Stimuli-responsive systems exploit local differences in pH, ROS, glutathione (GSH), or enzyme activity to control payload release (67). Sequential systems first modify vascular, stromal, or inflammatory barriers and then deliver cytotoxic or immunologically active cargo (68). Biomimetic coatings can reduce immune clearance and improve cellular targeting (69), whereas spatially directed systems extend delivery beyond the primary tumor to TDLNs and metastatic niches (70, 71). Multifunctional platforms combine two or more of these features with chemotherapy, radiotherapy, metabolic intervention, or physical modalities (72, 73). These approaches are not mutually exclusive; the relevant combination depends on the biological barrier being addressed. Accordingly, the following subsections retain a barrier-based organization while explicitly linking each barrier to the polymer-engineering principles most suited to overcome it. Section 3.1 focuses on stimuli-responsive ICD induction, antigen/adjuvant or STING co-delivery, and lymphoid-targeted immune priming; Section 3.2 emphasizes stromal normalization, FAP/MMP-responsive targeting, size-switching and sequential TME priming; Section 3.3 addresses cell-selective targeting, phenotype reprogramming and local hydrogel depots; and Section 3.4 highlights pH-, redox-, and hypoxia-responsive release together with metabolic modulation. **Figure 2** summarizes these barrier-to-design relationships and their therapeutic consequences. As most approaches remain preclinical, their translational potential should be evaluated in terms of biodistribution, safety, formulation complexity and manufacturability.

**Figure 2:**
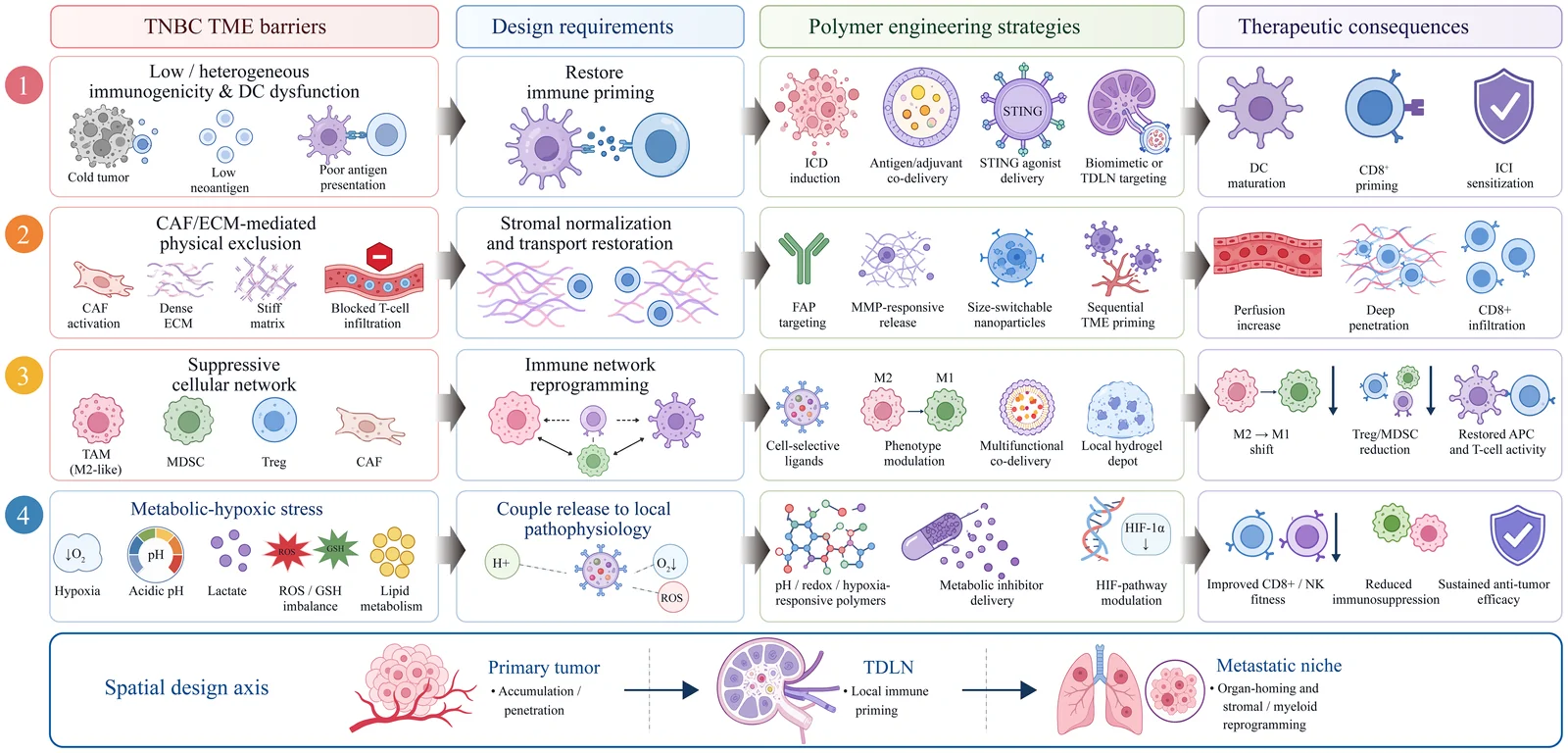
Barrier-to-design framework for polymer-based delivery systems in TNBC immunotherapy. Major TNBC TME barriers generate distinct delivery requirements, which are matched to stimuli-responsive activation, sequential TME priming, biomimetic or ligand-directed targeting, size-switchable/penetration-enhancing design, local hydrogel depots and multifunctional co-delivery. These engineering strategies converge on restoration of immune priming, stromal normalization, suppressive-cell reprogramming and metabolic/hypoxic relief. The lower spatial axis emphasizes that primary tumors, tumor-draining lymph nodes and metastatic niches require distinct physicochemical and pharmacological design criteria. The central principle is barrier matching rather than maximal material complexity.

### 3.1 Polymeric systems for enhancing tumor immunogenicity and antigen presentation

Within the TNBC TME, low TMB, limited neoantigen production, and DC dysfunction collectively contribute to inadequate tumor immunogenicity and impaired antigen presentation. These factors culminate in an “immune-cold” phenotype, a key factor in the limited efficacy of ICIs (74). PBDS can address these defects through controlled release and modular delivery to enhance TNBC immunogenicity. This is achieved by inducing ICD, delivering tumor antigens and adjuvants, and activating the cyclic GMP-AMP synthase–stimulator of interferon genes (cGAS-STING) pathway. Collectively, these mechanisms can restore the antigen-presenting function of DCs and facilitate the transformation of the immune environment from “cold” to “hot,” offering a promising strategy to overcome the challenges in TNBC immunotherapy (30).

Accordingly, PBDS that address insufficient immune priming primarily rely on four engineering logics: stimuli-responsive induction of ICD, synchronized co-delivery of tumor antigens with adjuvants or STING agonists, biomimetic or immune-cell-directed antigen presentation, and lymphatic/TDLN targeting. These strategies differ in where the cancer–immunity cycle is reinforced: ICD-generating systems increase antigen and danger-signal availability within the tumor; nanovaccines coordinate antigen protection with dendritic-cell activation; STING-oriented systems amplify innate sensing; and lymph-node-directed designs aim to spatially concentrate immune priming while limiting systemic inflammatory exposure (75, 76).

pH- and ROS-responsive polymeric nanoparticles, such as those based on poly(lactic-co-glycolic acid) (PLGA) or polyethyleneimine (PEI), loaded with chemotherapeutic drugs or photothermal/photodynamic agents, can stimulate tumor cells to release damage-associated molecular patterns (DAMPs), including calreticulin, high-mobility group box 1, and adenosine triphosphate (ATP). These DAMPs subsequently enhance uptake by DCs and facilitate antigen cross-presentation (77, 78). Shi et al. reviewed polymeric particle-based antigen delivery systems constructed from materials such as PLGA, chitosan, and PEI, highlighting the effects of particle size and surface modification on lymph-node targeting, antigen protection, controlled release, and DC activation (79). Yan et al. developed a personalized multi-epitope nanovaccine for TNBC, termed CM-CpG-aCD40, by integrating tumor cell membrane-derived antigens with the Toll-like receptor 9 (TLR9) agonist CpG and an agonistic anti-CD40 antibody. This nanoplatform accumulated in peripheral immune organs and coordinated antigen presentation with B cell- and CD8^+^ T cell-mediated antitumor immunity, thereby markedly enhancing therapeutic efficacy against TNBC. These findings suggest that co-delivery of tumor antigens and immunostimulatory adjuvants within nanovaccine systems may represent a promising strategy to overcome the limited immunogenicity and insufficient immune responsiveness of TNBC (80). Cationic polymers, including chitosan and PEI derivatives, when loaded with mRNA vaccines or STING agonists, facilitate cytoplasmic delivery and stimulate IFN production, thereby enhancing adaptive immune responses. Xu et al. developed a cationic fluoropolymer-based nanovaccine that efficiently delivered tumor membrane-derived antigens into DCs and promoted antigen cross-presentation. In an orthotopic postoperative 4T1 TNBC model, this strategy, combined with immune checkpoint blockade, markedly inhibited tumor recurrence and lung metastasis while inducing durable immune memory. These findings support the use of polymeric nanovaccines to enhance postoperative immunotherapy in TNBC (81). Recent cross-platform evidence further supports coordinated activation across the tumor–lymphoid axis. Ye et al. combined tumor-directed ICD induction with lymph-node immune activation using dual-pronged lipid nanocarriers in TNBC (82), whereas Xu et al. demonstrated that a minimalist self-assembled nanosystem could integrate multiple immune-activating mechanisms without excessive architectural complexity (83). Although these systems are not classical PBDS, they provide transferable principles for spatial immune activation and minimum-sufficient multifunctionality in polymer engineering.

Critical summary. PBDS that restore immunogenicity provide a direct route to initiate the cancer–immunity cycle, and polymeric co-delivery can synchronize antigen release with adjuvant or STING activation more effectively than uncoordinated free-drug combinations. However, this advantage is counterbalanced by strong model dependence, interpatient variability in antigen availability and baseline immune competence, and the narrow therapeutic window of innate immune agonists. Lymph-node targeting is additionally sensitive to particle size, surface chemistry and protein-corona formation. The next design priority should therefore be spatially restricted and biomarker-matched immune activation rather than maximal stimulation. Humanized models, patient-derived models incorporating immune components and longitudinal immune profiling will be necessary to determine whether these platforms can reproducibly convert immune-excluded TNBC into ICI-responsive disease (**Figure 3**; **Table 1**).

**Figure 3:**
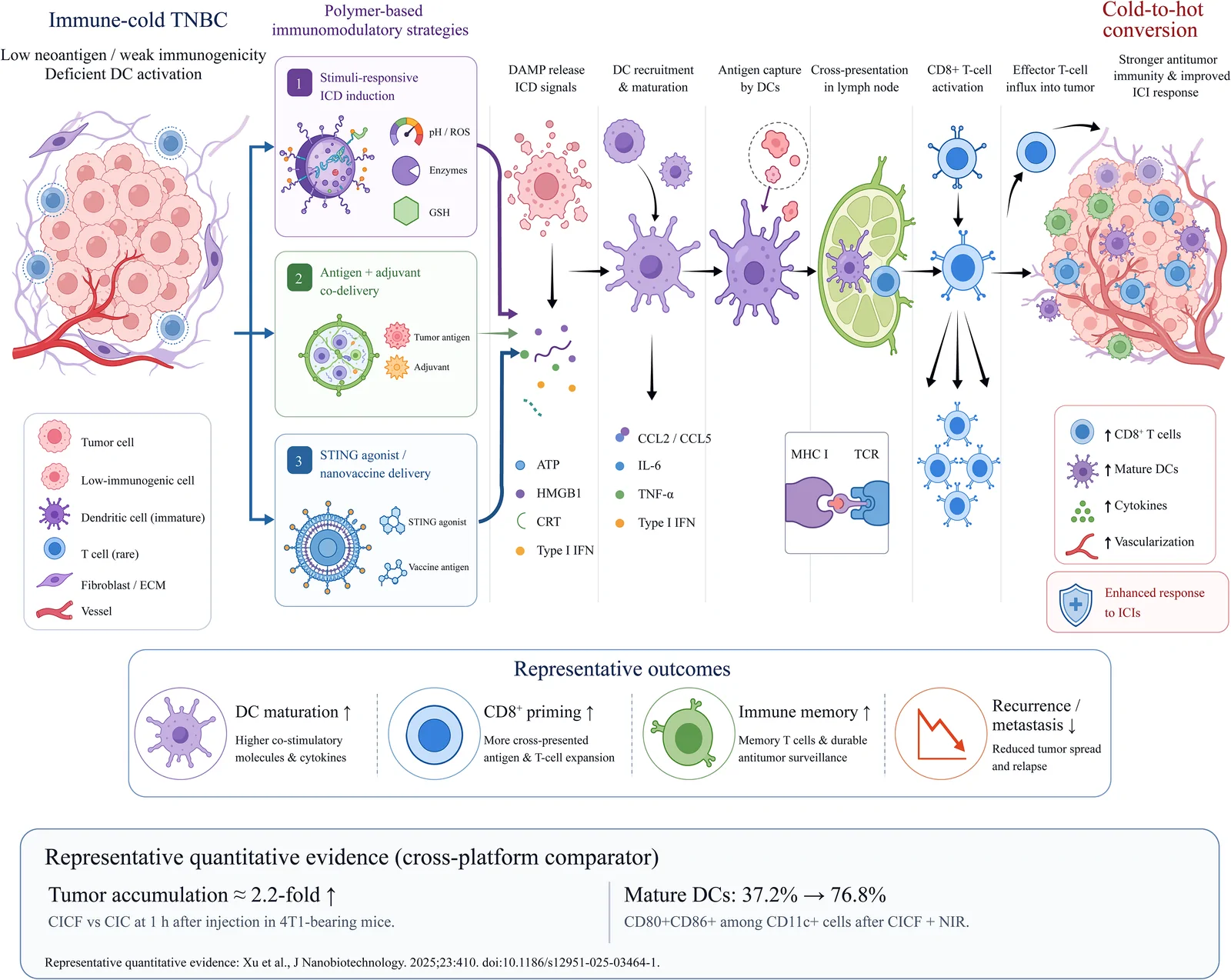
Restoring immunogenicity and antigen presentation through barrier-matched polymer design. Stimuli-responsive ICD, antigen/adjuvant co-delivery and STING-oriented delivery reinforce complementary steps of the cancer-immunity cycle, including DAMP release, dendritic-cell recruitment and maturation, antigen capture and cross-presentation, CD8^+^ T-cell priming and effector-cell influx. The overall design goal is conversion of immune-cold TNBC into a more immune-active state with improved responsiveness to immune checkpoint blockade. A representative recent cross-platform study is included as a quantitative comparator, showing approximately 2.2-fold higher tumoral fluorescence at 1 h and an increase in mature DCs from 37.2% to 76.8%. Data summarized from Xu et al., J Nanobiotechnology. 2025;23:410.

**Table 1:** Recent high-quality polymer-based systems for enhancing tumor immune response and antigen presentation in breast cancer/TNBC.

Polymeric platform	Payload / functional cargo	Design principle / immune target	Key findings / model	Main limitation	Ref.
PLGA particle vaccine	Antigen + Riboxxim	Innate activation + antigen delivery; TLR3/RIG-I	Strong antitumor immunity; synergy with ICB	Not TNBC-specific; model-dependent immune activation	(111)
DEC-205 peptide-modified PLGA nanovaccine	Antigen + CpG	DC-targeted antigen/adjuvant co-delivery	DC maturation, cross-presentation and antitumor immunity	DC-targeting efficiency and systemic innate toxicity require *in vivo* optimization	(112)
Polymeric micelles + polymersomal CpG	Gemcitabine + paclitaxel + CpG	ICD + sequential immune priming	Converted postoperative cold TNBC to immune-responsive TME	Complex multi-component formulation; mainly murine evidence	(113)
α5β1 integrin-targeted reduction-sensitive micelles	Paclitaxel	Ligand targeting + redox-responsive ICD	Selective ICD and improved chemo-immunotherapy	Target heterogeneity; translation of integrin targeting uncertain	(114)
PLGA-based local immunochemotherapy hydrogel	CpG ODN + gemcitabine + paclitaxel-loaded PLGA	Local depot + programmable sequential release	Local innate activation plus chemotherapy in TNBC	Requires accessible lesion/local administration; release reproducibility	(115)
Polymer-conjugated STING agonist nanoparticles	Cyclic dinucleotide STING agonist	Stimuli-sensitive polymer conjugation / innate sensing	Improved stability, loading, therapeutic window and immune memory	STING agonist inflammatory toxicity; cross-tumor evidence	(116)
Dual-STING-activating polymeric micelles	cGAMP + PC7A	Programmed burst + delayed STING activation	Expanded temporal window of STING immunotherapy	Timing/dose complexity; limited human validation	(117)
Tumor-responsive logic-gated STING nanomedicine	Dual pH/hypoxia-responsive polymeric nanoparticle	AND-gate tumor-selective activation	Reduced systemic toxicity with tumor-selective STING activation	Trigger heterogeneity can cause under/over-release	(118)
Polyoxazoline-based nanovaccine	Neoantigen peptides + TLR agonists + siRNA	Multifunctional antigen/adjuvant co-delivery	Enhanced antitumor immune response	Complex cargo selection and manufacturing; not TNBC-specific	(119)
Biomimetic personalized nanovaccine	Antigen-enriched tumor cell membrane nanovaccine	Biomimetic personalized antigen presentation	IFN-γ-enriched tumor cell membrane nanovaccine improved antigen presentation and early intervention potential	Personalized manufacturing and batch reproducibility	(120)
Artificial DC-like vesicle nanovaccine	Modified tumor-cell membrane vesicles	Biomimetic APC-like antigen presentation	Artificial dendritic-cell-like vesicles generated personalized autologous nanovaccines	Autologous production, QC and scale-up remain major barriers	(121)

### 3.2 Polymeric systems targeting stromal and ECM barriers

In TNBC, CAF-driven ECM remodeling is a major stromal mechanism underlying immunotherapy resistance. Excessive deposition of collagen, fibronectin, and hyaluronic acid increases matrix stiffness, compresses tumor vessels, and restricts both cytotoxic T-cell infiltration and the penetration of macromolecular immune therapeutics, thereby promoting an immune-excluded phenotype (84). Consequently, CAF/ECM-targeted PBDS should be viewed as both penetration-enhancing carriers and stromal immunomodulatory platforms that may improve TNBC response to ICB. From a design perspective, CAF/ECM-directed PBDS mainly use stromal normalization, FAP-directed targeting, MMP/enzyme-responsive or size-switchable penetration, and sequential TME-priming strategies to restore transport without indiscriminate matrix destruction (17, 76).

Panagi et al. found that tranilast-loaded polymeric micelles based on polyethylene glycol (PEG)-b-poly(benzyl-L-glutamate) effectively reprogrammed the mechanical TME in 4T1 and E0771 breast cancer models. This strategy reduced stromal stiffness, improved tumor perfusion, attenuated TGF-β-related immunosuppressive signaling, and increased T-cell infiltration. Notably, when combined with ICB-based nano-immunotherapy, tranilast micelles markedly enhanced antitumor efficacy, suggesting that stromal normalization can sensitize TNBC-like tumors to immunotherapy (85). Zhang et al. developed lipid poly-γ-glutamic acid (PGA)/PolyMet-pRLN nanoparticle (LPPR) and evaluated it in a desmoplastic 4T1 TNBC model. By downregulating CAF-related markers such as α-SMA, FAP, and TGF-β, LPPR promoted CD3^+^, CD4^+^, and CD8^+^ T-cell infiltration and enhanced the antitumor effect of anti-PD-L1 therapy. These findings support the concept that CAF-targeted PBDS can alleviate immune exclusion and overcome resistance to checkpoint blockade (86). Additionally, enzyme-responsive PBDS can improve immunotherapy by enhancing drug penetration through dense ECM barriers. He et al. developed an MMP-2-responsive, size-switchable nanosystem, UAMSN@Gel-PEG, in which ultrasmall amino-modified mesoporous silica nanoparticles were encapsulated within PEG-conjugated gelatin. Following accumulation in 4T1 tumors, MMP-2-mediated degradation of the gelatin shell exposed the ultrasmall nanoparticles, thereby facilitating their penetration into deeper tumor regions. Although this platform was originally designed for hydrogen therapy rather than immune checkpoint blockade, its enzyme-triggered size-switching strategy could potentially be adapted for the intratumoral delivery of immune adjuvants, immunogenic cell death-inducing agents, or checkpoint-blockade therapeutics in stroma-rich TNBC (87, 88). FAP-targeted polymeric systems represent another precise approach for CAF modulation. Furthermore, Gao et al. developed FAP single-chain antibody-decorated biomimetic PLGA nanoparticles, which restored activated CAFs to a quiescent state, promoted DC maturation, relieved lymphocyte suppression, and increased cytotoxic T-cell infiltration in breast cancer models (89, 90). More recently, Xian et al. developed a CAF-directed nano-regulator for TNBC that precisely targeted and modulated CAFs, reduced stromal barriers and enhanced chemo-immunotherapy, providing recent TNBC-specific evidence for selective stromal modulation (91).

Critical summary. The principal advantage of CAF/ECM-targeted PBDS is that they treat stromal transport failure as an actionable therapeutic barrier rather than as a passive feature of the TME. Stromal normalization can improve perfusion and immune-cell trafficking, whereas enzyme-responsive or size-switchable systems can enhance deep penetration without requiring global matrix depletion. Nevertheless, indiscriminate CAF ablation or extensive ECM degradation may disrupt tissue homeostasis, alter vascular integrity or remove fibroblast subsets with context-dependent tumor-restraining functions. Controlled normalization, CAF-subtype selection and temporally staged delivery are therefore preferable to nonspecific stromal destruction. Translational studies should quantify not only tumor shrinkage but also perfusion, matrix remodeling, immune infiltration, biodistribution and off-tumor stromal effects (**Figure 4**; **Table 2**).

**Figure 4:**
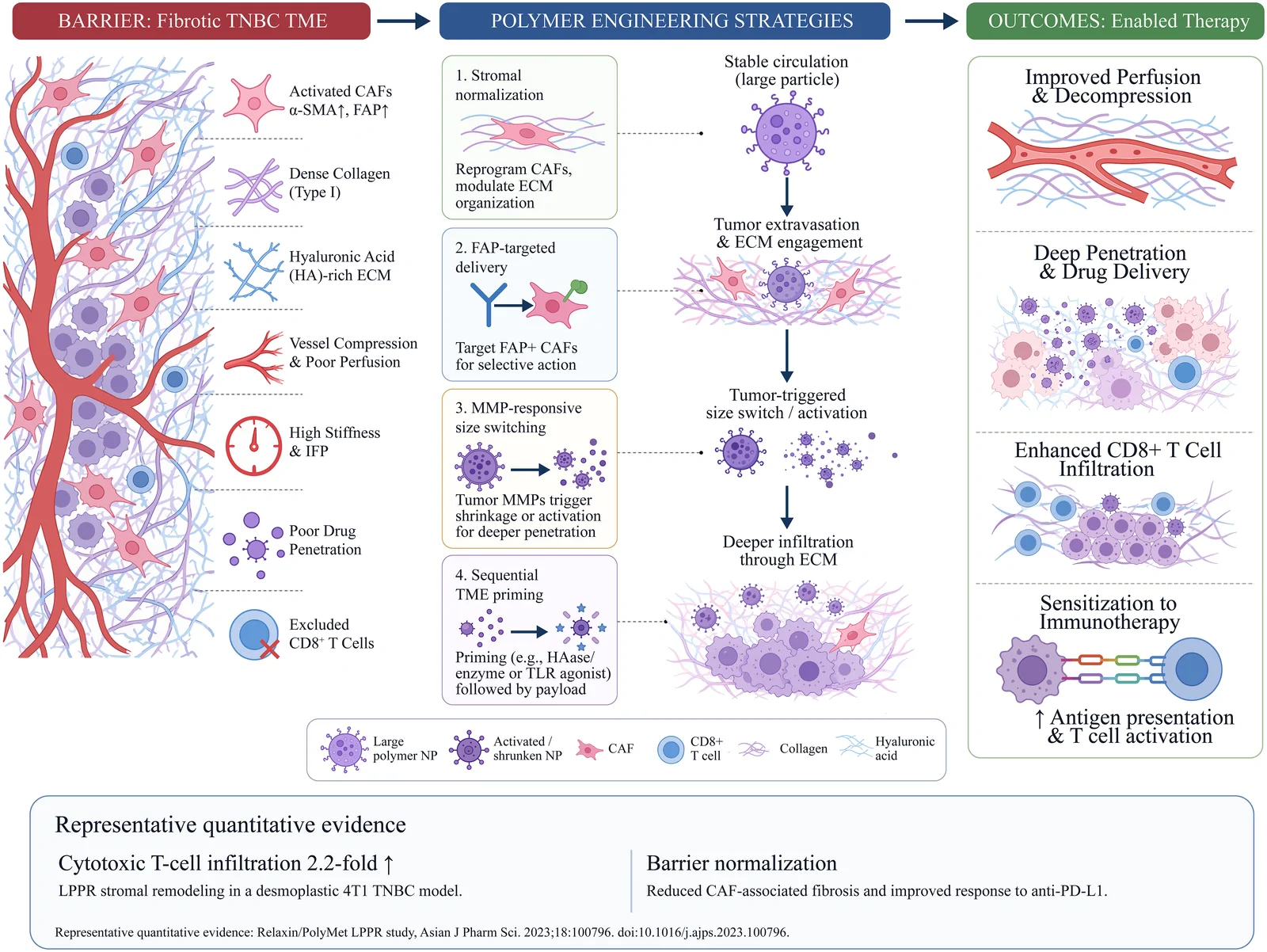
CAF/ECM barrier-adapted polymer design. The fibrotic TNBC TME, characterized by activated CAFs, dense collagen/HA-rich ECM, vessel compression, increased stiffness and interstitial fluid pressure, poor drug penetration and T-cell exclusion, creates a major transport barrier. Stromal normalization, FAP-directed targeting, MMP-responsive size switching and sequential TME priming are presented as complementary strategies to restore perfusion and deep penetration while enhancing CD8^+^ T-cell infiltration and immunotherapy sensitivity. Representative quantitative evidence from the LPPR study shows a 2.2-fold increase in cytotoxic T-cell infiltration after stromal remodeling. The key design principle is controlled stromal normalization rather than indiscriminate ECM depletion.

**Table 2:** Recent high-quality polymer-based systems targeting stromal and ECM barriers in TNBC.

Polymeric platform	Payload / functional cargo	Design principle / immune target	Key findings / model	Main limitation	Ref.
Tranilast-loaded PEG-PBLG micelles	Tranilast	Stromal normalization; CAF/stiffness modulation	Reduced stiffness and improved nano-immunotherapy in 4T1/E0771	Murine models; optimal degree/timing of stromal normalization uncertain	(85)
Pirfenidone-loaded polymeric micelles	Pirfenidone	Mechanical TME normalization	Restored biomechanical abnormalities at much lower dose; potentiated immunotherapy	Cross-tumor mouse models; long-term stromal effects unknown	(18)
Lipid PGA/PolyMet-pRLN nanoparticles	Relaxin plasmid	TGF-β/CAF reprogramming	Reduced CAF-mediated immunosuppression in 4T1 TNBC	Gene-delivery complexity; model dependence	(86)
FAP-targeting biomimetic PLGA nanoparticles	Calcipotriol	FAP-directed CAF normalization	Restored activated CAFs toward quiescence; enhanced antitumor immunity	FAP is not CAF-exclusive; off-tumor stromal targeting risk	(89)
Anti-FAP polymeric nanoparticles	8-O-methylfusarubin	FAP+ CAF targeting	Selective CAF targeting and improved antitumor activity	Primarily preclinical; FAP heterogeneity	(90)
Anti-FAP@OMF nanoparticles + CAR-T	8-O-methylfusarubin	CAF targeting + cellular immunotherapy	Enhanced CAR-T cytotoxicity in TNBC–CAF heterospheroids	Complex combination; limited *in vivo*/clinical validation	(122)
Gelatin MMP-2/photothermal-responsive nanoparticles	DOX + ICG	MMP-responsive release / penetration	Improved penetration and on-demand therapy	Not designed primarily for immunotherapy; trigger heterogeneity	(123)
Dual pH/GSH-responsive collagen-barrier-overcoming nanoparticles	DOX	Dual-responsive release + collagen barrier modulation	Improved penetration across dense collagen	Enzyme/collagen manipulation may affect normal stroma	(124)
ECM-targeted photodynamic immunomodulator	PpIX + SIS3	ECM remodeling + CAF reprogramming	Improved photodynamic immunotherapy in fibrotic breast cancer	Multimodal complexity; light penetration constraints	(125)
Collagen-binding hybrid nanocarrier	Paclitaxel	Collagen binding / local retention	Enhanced local retention for intraductal treatment	Local route; not systemic TNBC immunotherapy	(126)
HA–chitosan-conjugated PLGA nanoparticles	DOX + ICG	CD44 targeting + pH-responsive penetration	Improved targeted chemo-photothermal TNBC therapy	CD44 heterogeneity; photothermal translation constraints	(127)
PLGA-based rhein/hesperidin nanoparticles	Rhein + hesperidin	CAF/CAA/CCL2 immune-stromal remodeling	Reduced suppressive cells; increased cytotoxic T cells	Complex stromal/adipocyte mechanism; mainly murine evidence	(96)
Precision CAF nano-regulator (TET@ACNP)	Tetrandrine	CAF membrane/anisamide targeting + CAF modulation	Recent TNBC-specific CAF nanoregulation and chemo-immunotherapy	New preclinical study; long-term off-tumor stromal safety unknown	(91)

### 3.3 Polymeric delivery systems targeting immunosuppressive cellular components

Although effective antigen presentation is essential for initiating antitumor immunity, a complex network of TAMs, MDSCs, Tregs, and suppressive CAFs sustains immunosuppression within the TNBC TME. Targeting immunosuppressive cell populations within the TME of TNBC using engineered polymeric delivery systems has emerged as a pivotal strategy to enhance antitumor immune responses. Unlike earlier approaches that focused on cell depletion, contemporary research prioritizes reprogramming immune cell phenotypes through precision delivery, aiming to reverse, rather than merely eliminate, critical cell populations within the TME. Here, the dominant engineering logics are cell-selective targeting, phenotype reprogramming, feedback-responsive release and local depot delivery, with the goal of disrupting cooperative TAM-MDSC-Treg-CAF circuits rather than depleting a single cell population.

Yazdimamaghani et al. demonstrated that polymeric micellar nanoparticles co-delivering a Toll-like receptor 7/8 (TLR7/8) agonist (R848) and a phosphoinositide 3-kinase delta (PI3Kδ) inhibitor effectively reprogrammed the TME immune response. In an orthotopic breast cancer model, this system facilitated TAM polarization toward the M1 phenotype while concurrently reducing MDSC accumulation and the proportion of tissue-resident Tregs. When used in conjunction with local radiotherapy, this approach significantly enhanced adaptive T and B cell immunity, resulting in superior tumor suppression compared to radiotherapy alone (92). Similarly, Li et al. illustrated that inhibition of complement component 5a receptor 1 (C5aR1) successfully reprograms TAMs, thereby reversing resistance to PARP inhibitors in breast cancer. This finding highlights the critical role of myeloid cells as key determinants of therapeutic response within the TME (48). In addition, Dong et al. developed M2-targeting peptide-modified chitosan–curcumin nanoparticles (M2pep-Cs-Cur NPs), which promoted TAM reprogramming toward an antitumor phenotype and suppressed TNBC cell proliferation and invasion *in vitro*. Combination with anti-PD-L1 further improved tumor control in an orthotopic TNBC model (93). Zhang et al. developed a redox-responsive polymeric nanoplatform based on hyaluronic acid-conjugated disulfide-linked polyethyleneimine for the combined regulation of tumor cells and TAMs. This platform promoted TAM repolarization, alleviated the immunosuppressive TME, and enhanced antitumor efficacy in breast cancer models. However, its effects require further validation in immunologically heterogeneous human TNBC models (94). Song et al. developed a self-augmented ROS-responsive polymeric nanodrug co-delivering paclitaxel and the indoleamine 2,3-dioxygenase 1 (IDO1) inhibitor 1-methyltryptophan (1-MT) for TNBC chemoimmunotherapy. In MDA-MB-231 and 4T1 models, this nanoplatform promoted ROS-triggered drug release and paclitaxel-induced ICD, while IDO1 blockade alleviated the immunosuppressive TME by reducing Tregs and M2-like TAM infiltration. As a result, the system enhanced antitumor immunity and suppressed both primary tumor growth and lung metastasis (95). Furthermore, Huang et al. engineered nanoparticles co-loaded with rhein and hesperidin using PLGA. These nanoparticles were demonstrated to inhibit CAF activation and CCL2 secretion from cancer-associated adipocytes. Consequently, they remodeled the TME immune landscape, reduced Treg infiltration, and promoted the accumulation of cytotoxic T cells, thereby demonstrating safe and potent antitumor efficacy in TNBC models (96).

Local polymeric hydrogels provide a complementary approach for modulating suppressive cellular networks by prolonging intratumoral drug retention while limiting systemic exposure. Mei et al. developed an injectable, weak-acid-responsive polymetformin hydrogel incorporating violet phosphorus nanosheets co-loaded with methylene blue and doxorubicin together with anti-PD-L1. Following intratumoral administration, the system combined photodynamic therapy, chemotherapy, and checkpoint blockade to remodel the TNBC TME and suppress primary and abscopal tumor progression and metastasis (97). In a subsequent study, Mei et al. developed a pH- and thermo-responsive drug-formed hydrogel consisting of JQ1-loaded graphdiyne oxide nanosheets, polymetformin, and dibenzaldehyde-functionalized PEG. Combined with photothermal therapy, this system inhibited primary and abscopal tumors, promoted long-term immune memory, and reduced metastatic dissemination (98). These studies illustrate how local hydrogel depots can provide sustained or stimulus-responsive release while integrating local TME modulation with immunotherapy.

Critical summary. Cell-directed PBDS can reprogram TAMs and related suppressive populations with lower systemic exposure than untargeted immunomodulators, but single-cell targeting rarely captures the cooperative TAM–MDSC–Treg–CAF network that sustains TNBC immune escape. Multi-pathway platforms therefore have a stronger mechanistic rationale, yet each additional ligand, payload or release module increases pharmacokinetic mismatch, manufacturing complexity and quality-control burden. Local hydrogel depots can partly mitigate this trade-off by prolonging local exposure and enabling staged release, but their clinical use is constrained by lesion accessibility and the need for reproducible degradation and release kinetics. Future designs should prioritize the minimum sufficient combination of modules needed to disrupt the dominant suppressive circuit in a biomarker-defined tumor (**Figure 5**; **Table 3**).

**Figure 5:**
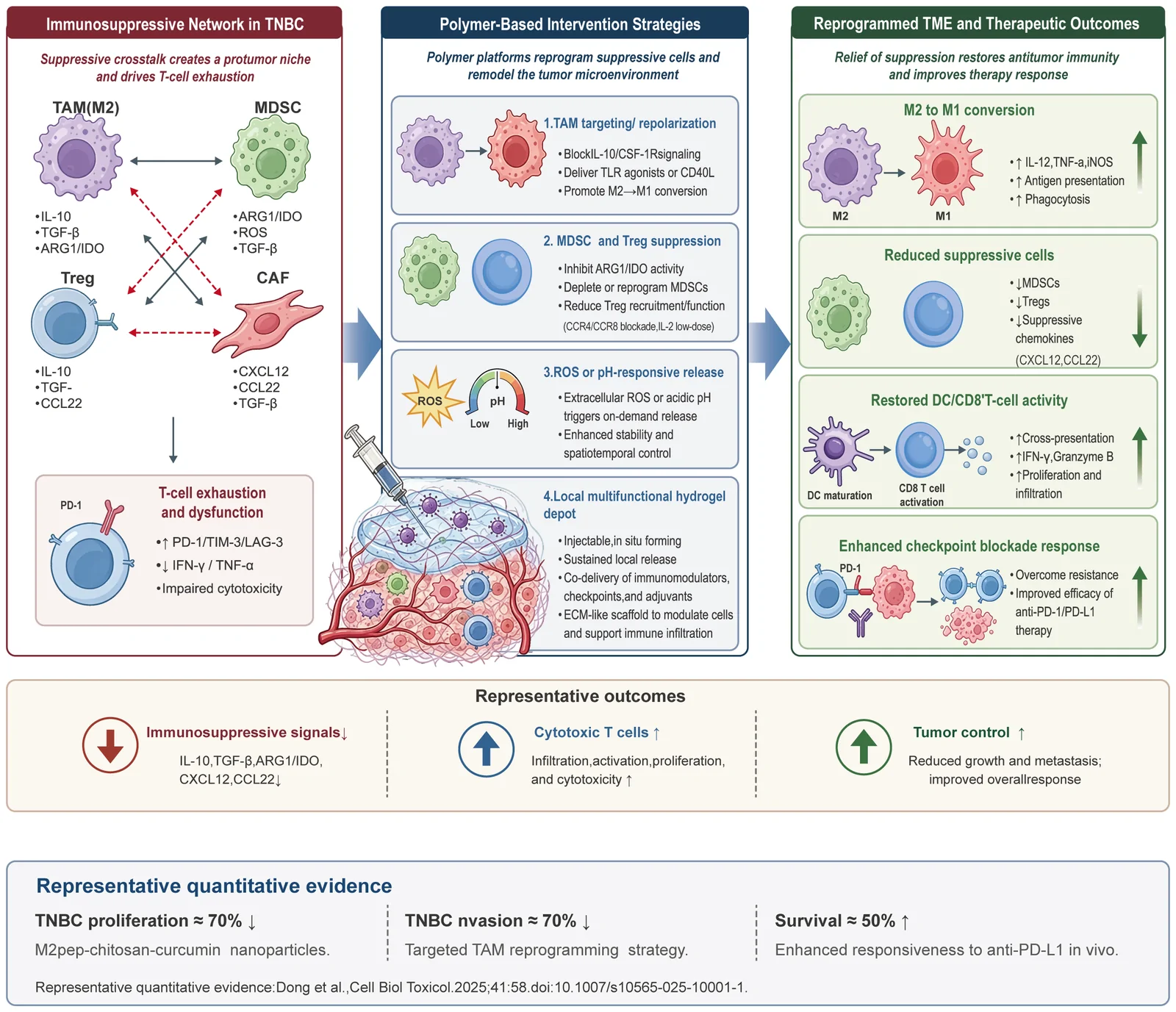
Reprogramming suppressive cellular networks using targeted PBDS and local hydrogel depots. TAMs, MDSCs, Tregs and CAFs form reciprocal suppressive circuits that promote T-cell exhaustion. Cell-selective targeting, phenotype reprogramming, ROS/pH-responsive release and local multifunctional hydrogels can reduce suppressive signaling, promote M2-to-M1 repolarization, restore dendritic-cell/CD8^+^ T-cell activity and enhance checkpoint-blockade response. Representative quantitative evidence from M2pep-chitosan-curcumin nanoparticles shows approximately 70% inhibition of TNBC cell proliferation, approximately 70% inhibition of invasion and approximately 50% survival enhancement with improved anti-PD-L1 responsiveness.

**Table 3:** Recent high-quality polymeric delivery systems targeting immunosuppressive cellular components in breast cancer/TNBC.

Polymeric platform	Payload / functional cargo	Design principle / immune target	Key findings / model	Main limitation	Ref.
M2pep–chitosan nanoparticles	Curcumin	M2-TAM targeting + phenotype reprogramming	~70% proliferation/invasion inhibition; improved anti-PD-L1 response	Single-cell targeting; mouse-model translation	(93)
M1 macrophage membrane-engineered PLGA nanoparticles	CDN + R848	Biomimetic targeting + TAM reprogramming	Shifted M2 toward M1 phenotype	Membrane-source standardization and QC	(128)
Redox-responsive R848 polymer nanogel	R848 prodrug	Redox-responsive innate activation / APC maturation	APC maturation and M2 repolarization with reduced systemic toxicity	TLR7/8 inflammatory risk; trigger variability	(129)
Polymeric nanocapsules	poly(I:C) + R848	HA/mannose targeting + dual innate agonists	Improved TAM targeting and M1-like activity	Dual-agonist systemic inflammation; not TNBC-specific	(130)
pH-responsive polymeric micelles	DOX + imiquimod	pH-triggered ligand exposure + immune activation	M1 polarization and immune activation	Acidic-pH heterogeneity; multimodal PK	(131)
LMWH–TOS polymeric micelles	DOX + imiquimod + anti-PD-L1	Chemoimmunotherapy + checkpoint blockade	Suppressed primary tumors and lung metastasis	Three-component regimen; formulation/PK complexity	(132)
POx-based polymeric micelles	Paclitaxel + PLX3397	CSF1R+ macrophage modulation + chemotherapy	Enhanced adaptive T-cell immunity in TNBC	CSF1R systemic effects; mouse evidence	(133)
Hesperidin-loaded PLGA nanoparticles	Hesperidin	Myeloid/Treg/CAA network remodeling	M2/Treg/MDSC ↓; CD8/DC ↑	Broad network effects; mechanism and human relevance need validation	(134)
PLGA rhein/hesperidin nanoparticles	Rhein + hesperidin	CAF/CAA-driven immune suppression	Cytotoxic T-cell infiltration ↑	Multi-target attribution difficult	(96)
Semiconducting biomimetic nanoparticles	PFODBT + atovaquone + TMP195	Hypoxia relief + TAM reprogramming	Shifted TAMs M2→M1	High multifunctional/material complexity	(135)
Redox-activatable sequential-release nanoparticles	Metabolic inhibitor + CRISPR/Cas9	Sequential metabolic/gene editing TIME reprogramming	Reversed immune escape	Gene-editing safety and CMC complexity	(136)
Violet-phosphorus/polymetformin immunogel	VPN/MB/DOX + anti-PD-L1	Local weak-acid-responsive hydrogel + multimodal immune activation	Primary/abscopal control and metastasis suppression	Intratumoral accessibility; multi-component manufacturing	(97)
Graphdiyne oxide/JQ1/polymetformin hydrogel	JQ1-GDYO + polymetformin	Dual-responsive local hydrogel + photothermal immunotherapy	Primary/abscopal inhibition, immune memory and reduced metastasis	Local administration; photothermal and formulation complexity	(98)

### 3.4 Polymeric delivery systems for metabolic and hypoxia-responsive TME reprogramming

Metabolic and hypoxic abnormalities in TNBC can also serve as delivery cues. PBDS responsive to extracellular acidity, redox conditions, ROS, or hypoxia can localize payload release, but these features are best viewed as triggers rather than therapeutic endpoints. Their therapeutic value depends on the cargo used to inhibit tumor metabolism, modulate immune checkpoints, or relieve hypoxia (99, 100).

Ren et al. developed a TME-activatable aptamer–polymer nanoassembly, DNA-PAE@BAY-876, for metabolic–immune reprogramming in TNBC. This system co-assembled PAE-modified PD-L1- and cytotoxic T-lymphocyte-associated protein 4 (CTLA-4)-antagonizing aptamers with the glucose transporter 1 (GLUT1) inhibitor BAY-876, enabling acidic TME-triggered release at tumor sites. By selectively suppressing glycolysis in TNBC cells, DNA-PAE@BAY-876 reduced PD-L1 N-linked glycosylation and attenuated Treg-mediated immunosuppression, thereby enhancing the efficacy of immune checkpoint blockade. In 4T1 and MDA-MB-231 TNBC models, this nanoassembly increased infiltration of CD8^+^ T cells and other antitumor immune populations, elevated IFN-γ and TNF-α levels, and produced superior tumor inhibition compared with conventional immune checkpoint antibody combinations (101). In a related study, Zhang et al. developed pH-sensitive nanomicelles composed of mPEG-hyd-PLGA for the co-delivery of paclitaxel, the C-X-C motif chemokine receptor 4 (CXCR4) antagonist AMD3100, and the small-molecule PD-1/PD-L1 inhibitor BMS-1. In a 4T1 orthotopic TNBC model, this platform reduced CAF activation, angiogenesis, and extracellular matrix deposition, while limiting the recruitment of immunosuppressive cells and enhancing CD8^+^ T-cell infiltration. These effects collectively inhibited primary tumor growth and distant metastasis. Nevertheless, the efficacy of this system has thus far been demonstrated primarily in murine models, and its pharmacokinetic behavior, long-term safety, and therapeutic reproducibility require further evaluation (102). Liu et al. developed hypoxia-responsive polymeric micelles for the co-delivery of doxorubicin and HIF-1α-targeting short hairpin RNA (shRNA) in TNBC. The micelles were constructed using an azo-linked methoxy poly(ethylene glycol)–poly(L-lysine) (mPEG–PLL) copolymer, enabling hypoxia-triggered disassembly and rapid payload release within hypoxic tumor regions. In TNBC models, this system efficiently suppressed HIF-1α and its downstream signaling, remodeled the hypoxic TME, and enhanced chemotherapy response, thereby synergistically inhibiting primary tumor growth and distant metastasis. This study emphasizes hypoxia-responsive polymeric co-delivery as a promising strategy to overcome HIF-1α-mediated therapeutic resistance in TNBC (103). Cross-platform systems based on Prussian blue and other bioactive metallic nanoparticles similarly illustrate how intrinsic catalytic or redox activity can be coupled to immune modulation. For PBDS, the transferable lesson is that TME-responsive chemistry should be incorporated only when the responsive module provides a measurable gain in spatial control or therapeutic index (104, 105).

Critical summary. Metabolic- and hypoxia-responsive PBDS are attractive because they couple drug release to pathophysiological features that directly suppress antitumor immunity. Their main limitation is that pH, redox state, oxygen tension and metabolic dependencies vary spatially within the same tumor and temporally during treatment; a response threshold optimized in one model may therefore under-release or prematurely release cargo in another. Protein-corona formation and altered perfusion under hypoxia can further decouple *in vitro* responsiveness from *in vivo* delivery. Future systems should pair responsive chemistry with measurable companion biomarkers, define activation thresholds quantitatively and avoid excessive multifunctionality unless each module contributes demonstrable incremental benefit over a simpler formulation (**Figure 6**; **Table 4**).

**Figure 6:**
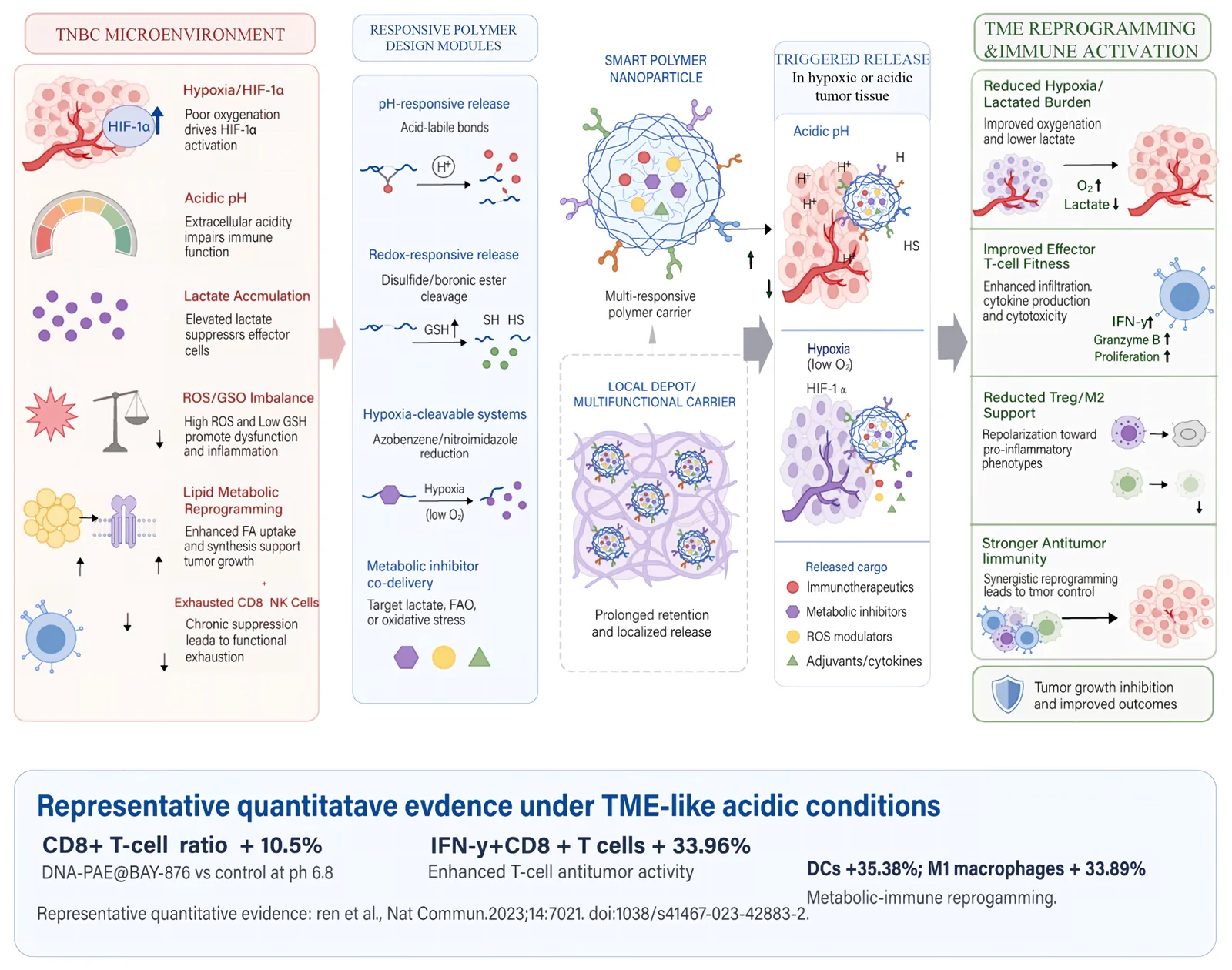
Metabolic- and hypoxia-responsive TME reprogramming. Hypoxia/HIF-1α signaling, extracellular acidity, lactate accumulation, redox imbalance and lipid-metabolic rewiring impair effector lymphocyte function in TNBC. pH-responsive, redox-responsive and hypoxia-cleavable polymers, together with metabolic-inhibitor co-delivery and local multifunctional depots, enable spatially restricted cargo release and metabolic–immune reprogramming. Representative quantitative evidence for the TME-activatable DNA-PAE@BAY-876 system under pH 6.8 shows increases in the CD8^+^ T-cell ratio (+10.5%), IFN-γ+ CD8^+^ T cells (+33.96%), DC frequency (+35.38%) and M1 macrophage frequency (+33.89%) compared with control conditions.

**Table 4:** Recent high-quality polymeric delivery systems for metabolic and hypoxia-responsive TME reprogramming in breast cancer/TNBC.

Polymeric platform	Payload / functional cargo	Design principle / immune target	Key findings / model	Main limitation	Ref.
Hypoxia-responsive mPEG-PLL-AZO micelles	DOX + HIF-1α shRNA	Hypoxia-cleavable release + HIF-1α inhibition	Synergistic chemo-gene therapy in TNBC	Hypoxia heterogeneity; nucleic-acid delivery complexity	(103)
Tumor hypoxia-activated polymeric micelles	Hypoxia-activated immunogenic cargo	Hypoxia-sensing release	Triggered systemic antitumor immunity	Activation threshold may vary among lesions	(137)
Sequential-release nanomaterial	HIF-1α siRNA + chemotherapy	Sequential hypoxia/HIF control	Relieved hypoxia-mediated resistance	Multi-payload release synchronization	(138)
GSH-responsive chitosan micelles	NO donor + DOX	Redox-responsive release + hypoxia relief	Improved chemotherapy/metastasis inhibition	Redox/NO control and systemic effects	(19)
Biotin-functionalized polymeric nanoparticles	CPT1-targeting system	FAO targeting / metabolic modulation	Targeted CPT1-mediated FAO driver of TNBC metastasis	Metabolic plasticity and subtype dependence	(139)
Multifunctional iPR@M1/Se nanoparticles	Lonidamine prodrug + Senaparib	Glycolysis + DDR + cGAS/STING	Metabolic reprogramming and immune activation	Highly multifunctional; manufacturing/PK complexity	(140)
Redox-activatable sequential-release nanoparticles	Metabolic inhibitor + CRISPR/Cas9	Dual metabolic inhibition + gene editing	Reversed metabolic heterogeneity/TIME	Gene-editing safety; CMC burden	(136)
CXCR4 antagonist peptide–docetaxel nanoparticles	Docetaxel	Metastatic-niche/CXCR4 targeting	Inhibited multi-organ metastasis	CXCR4 heterogeneity; not primarily immune-responsive	(141)
Hypoxia-responsive stereocomplex PEG-PLA micelles	Anticancer drug	Hypoxia-responsive delivery to metastatic tumor	Improved loading; reduced primary/metastatic growth	Older preclinical evidence; limited immune endpoints	(142)
HA–chitosan-conjugated PLGA nanoparticles	DOX + ICG	pH-sensitive release + photothermal stress	Targeted therapy-resistant TNBC microenvironment	Photothermal and CD44 heterogeneity	(127)
TPGS polymeric nanoparticles	PI3Kα inhibitors R7/R11	Pathway-focused delivery	Enhanced anticancer activity of PI3Kα inhibitors	Cell-line evidence; immune mechanism limited	(143)
Macauba oil-loaded polymeric micelles	Macauba pulp oil	Metabolic/migration modulation	Reduced TNBC migration/proliferation	Primarily *in vitro*; unclear immunotherapy relevance	(144)

## 4 Discussion and perspectives

### 4.1 Subtype- and biomarker-informed PBDS design

The molecular and spatial heterogeneity of TNBC argues against a universal PBDS formulation. Importantly, the representative PBDS studies summarized in **Tables 1**–**4** rarely stratified models prospectively according to BL1, BL2, mesenchymal (M), or LAR subtypes. Therefore, subtype–design relationships should be considered hypothesis-generating rather than clinically validated. BL1 tumors, which are frequently enriched for proliferative and DNA-damage-response programs, may be suitable for polymeric strategies that coordinate DNA-damaging or ICD-inducing cargo with immune activation. BL2 tumors show more heterogeneous growth-factor and metabolic programs, suggesting greater dependence on biomarker-defined pathway selection. Mesenchymal TNBC provides a rationale for CAF/ECM-normalizing, FAP-targeted or penetration-enhancing PBDS, whereas LAR tumors may warrant designs that integrate metabolic modulation with immune priming (106). However, molecular subtype alone is insufficient: spatial immune infiltration, CAF/ECM density, hypoxia and metabolic state should be incorporated as companion TME biomarkers (107). Thus, future clinical translation should move from subtype labels alone toward combined molecular–microenvironmental stratification (**Table 5**).

**Table 5:** Subtype- and TME-informed considerations for PBDS design in TNBC.

TNBC subtype	Key biological features	TME / delivery implications	PBDS design considerations	Ref.
BL1	High proliferation with enrichment of cell-cycle and DNA-damage-response programs.	Immune competence is variable; benefit from immune activation should not be assumed from subtype alone.	ICD- or DNA-damage-oriented payloads with immune co-stimulation when HRD and immune biomarkers support this strategy.	(145)
BL2	Growth-factor and metabolic signaling enrichment with marked biological heterogeneity.	Relevant barriers are likely pathway- and patient-dependent; stromal and metabolic states may vary.	Biomarker-selected pathway/metabolic cargo; stimuli-responsive release only when the corresponding trigger is demonstrated.	(146)
Mesenchymal (M)	EMT, motility, invasion and stemness-related programs.	CAF/ECM-rich and immune-excluded features may impair perfusion, penetration and T-cell trafficking.	Stromal normalization, FAP/MMP-informed targeting, or penetration-enhancing/size-switchable systems.	(147)
LAR	AR/luminal programs with PI3K-pathway and metabolic alterations in subsets.	Immune state is variable; pathway dependence may be more informative than the subtype label alone.	AR/PI3K/metabolic cargo; add immune priming only when a suppressive or immune-cold phenotype is documented.	(148)

These relationships are hypothesis-generating and should not be interpreted as established subtype-specific recommendations. PBDS selection should integrate molecular subtype with measurable TME features and target expression.

### 4.2 From multifunctionality to minimum sufficient complexity

Multifunctional PBDS can simultaneously address tumor killing, stromal normalization, immune-cell reprogramming and metabolic suppression, but increasing functional complexity does not automatically improve clinical value. Multiple payloads can have mismatched pharmacokinetics and release kinetics, while additional ligands, linkers and responsive motifs increase protein-corona variability, degradation products, batch-to-batch variability, storage and scale-up requirements, and regulatory burden (17). A practical design principle is therefore minimum sufficient complexity: each added module should solve a defined biological barrier and demonstrate incremental benefit over a simpler comparator. Early development should prioritize compositionally defined materials, orthogonal release assays, stability-indicating tests, and quality-by-design manufacturing rather than maximizing the number of functions (108).

### 4.3 Spatially resolved PBDS design: primary tumor, TDLN and metastatic niche

Spatial location changes the delivery problem. For primary tumors, PBDS must balance systemic stability with vascular access, stromal penetration, cellular uptake and intratumoral release. For TDLNs, lymphatic access, particle size/deformability, APC uptake, retention and spatially restricted immunostimulation become dominant (75). For pre-metastatic or established metastatic niches, prolonged circulation, organ-selective homing and reprogramming of myeloid/stromal components may be more important than high exposure in the primary lesion. These distinctions argue against assuming that a formulation optimized for the primary tumor will automatically perform well in TDLNs or distant niches. Spatially resolved biodistribution and efficacy should therefore be built into preclinical study design (109).

### 4.4 A clinically oriented translation roadmap

Clinical translation should pair each known barrier with a predefined validation strategy. TNBC heterogeneity requires biomarker-guided selection and testing across complementary models, including syngeneic models, humanized systems, patient-derived xenografts/organoids and, where feasible, tumor-on-chip platforms. Protein-corona uncertainty should be addressed using human biofluids and predefined pharmacokinetic/biodistribution assays rather than relying only on serum-free or murine *in vitro* testing (110). Biosafety programs should quantify degradation, organ retention, immune toxicity and repeated-dose effects. Manufacturing development should favor simplified formulations, scalable unit operations, batch reproducibility, stability-indicating assays and GMP-compatible critical quality attributes. Finally, TME-targeted PBDS are more likely to be clinically useful as adjuncts to established TNBC therapies when the polymer component provides a measurable clinical advantage—such as improved local exposure, TME priming or reduced systemic toxicity—rather than as stand-alone replacements (17).

## 5 Conclusion

TNBC immunotherapy is constrained not by a single defect but by interconnected immune, stromal and metabolic barriers that limit antigen presentation, immune-cell infiltration, drug penetration and effector function. PBDS provide a flexible means to address these barriers, but their value depends on rational barrier matching rather than on increasing carrier complexity. The most promising strategies combine spatially controlled immune activation, stromal normalization, selective reprogramming of suppressive cells and metabolically informed release while maintaining acceptable pharmacokinetics, biosafety and manufacturability. Future progress will require subtype- and biomarker-informed patient selection, clinically relevant models that capture TNBC heterogeneity, quantitative assessment of protein-corona and biodistribution effects, and quality-by-design manufacturing. Distinct design criteria should also be considered for primary tumors, TDLNs and metastatic niches. Ultimately, clinically useful PBDS are likely to emerge as precisely engineered partners for established TNBC therapies, providing a defined microenvironmental or delivery advantage while avoiding unnecessary multifunctional complexity.

## Glossary

1-MT: 1-methyltryptophan
ADCs: antibody-drug conjugates
ATP: adenosine triphosphate
BRCA1/2: breast cancer susceptibility gene 1/2
C5aR1: complement component 5a receptor 1
CAFs: cancer-associated fibroblasts
CCL2: C-C motif chemokine ligand 2
CCL22: C-C motif chemokine ligand 22
CD3: cluster of differentiation 3
cGAS-STING: cyclic GMP-AMP synthase–stimulator of interferon genes
CGRP: calcitonin gene-related peptide
CM-CpG-aCD40: cell membrane-derived antigen/CpG/anti-CD40 antibody nanovaccine
CpG: cytosine-phosphate-guanine
CSCs: cancer stem cells
CSF1R: colony-stimulating factor 1 receptor
CTLA-4: cytotoxic T-lymphocyte-associated protein 4
CXCL12: C-X-C motif chemokine ligand 12
CXCR4: C-X-C chemokine receptor type 4
DAMPs: damage-associated molecular patterns
DCs: dendritic cells
DNA: deoxyribonucleic acid
ECM: extracellular matrix
EGFR: epidermal growth factor receptor
EPR: enhanced permeability and retention
FAO: fatty acid oxidation
FAP: fibroblast activation protein
FASN: fatty acid synthase
GLUT1: glucose transporter 1
GSH: glutathione
H3K27ac: histone H3 lysine 27 acetylation
H3K27me3: histone H3 lysine 27 trimethylation
HDAC1: histone deacetylase 1
HER2: human epidermal growth factor receptor 2
HIF-1α: hypoxia-inducible factor 1 alpha
HIF-2α: hypoxia-inducible factor 2 alpha
HR+: hormone receptor-positive
ICB: immune checkpoint blockade
ICD: immunogenic cell death
ICIs: immune checkpoint inhibitors
IDO1: indoleamine 2,3-dioxygenase 1
IFN: interferon
IFN-γ: interferon-gamma
IL-6: interleukin-6
IL-10: interleukin-10
IL-12: interleukin-12
LDs: lipid droplets
LPPR: lipid poly-γ-glutamic acid/PolyMet-pRLN nanoparticle
MDSCs: myeloid-derived suppressor cells
MHC: major histocompatibility complex
MHC-II: major histocompatibility complex class II
MMP: matrix metalloproteinase
MMP-2: matrix metalloproteinase-2
mPEG: methoxy polyethylene glycol
mPEG-hyd-PLGA: methoxy polyethylene glycol-hydrazone-poly(lactic-co-glycolic acid)
mRNA: messenger RNA
myoCAF: myofibroblastic cancer-associated fibroblast
NADPH: nicotinamide adenine dinucleotide phosphate
NK: natural killer
NRF2: nuclear factor erythroid 2-related factor 2
ORR: objective response rate
OS: overall survival
OVOL2: ovo-like zinc finger 2
PAE: Poly(β-amino ester)
PARP: poly(ADP-ribose) polymerase
PBDS: polymer-based drug delivery systems
PD-1: programmed cell death protein 1
PD-L1: programmed death-ligand 1
PEG: polyethylene glycol
PEI: polyethyleneimine
PGA: poly-γ-glutamic acid
PI3Kδ: phosphoinositide 3-kinase delta
PLGA: poly(lactic-co-glycolic acid)
PLL: poly-L-lysine
PPAR: peroxisome proliferator-activated receptor
PRC2: polycomb repressive complex 2
PRMT1: protein arginine methyltransferase 1
PTEN: phosphatase and tensin homolog
RAMP1: receptor activity-modifying protein 1
ROS: reactive oxygen species
scRNA-seq: single-cell RNA sequencing
shRNA: short hairpin RNA
STING: stimulator of interferon genes
TAMs: tumor-associated macrophages
TDLNs: tumor-draining lymph nodes
TGF-β: transforming growth factor-beta
TLR7/8: Toll-like receptor 7/8
TLR9: Toll-like receptor 9
TMB: tumor mutational burden
TMB-H: tumor mutational burden-high
TMB-L: tumor mutational burden-low
TME: tumor microenvironment
TNBC: triple-negative breast cancer
TNF: tumor necrosis factor
TNF-α: tumor necrosis factor-alpha
Tregs: regulatory T cells
VEGF: vascular endothelial growth factor
α-SMA: alpha-smooth muscle actin
