# Correction: Advancements in polymer-based drug delivery systems targeting the tumor microenvironment for immunotherapy of triple-negative breast cancer

**DOI:** 10.3389/fimmu.2026.2016911

**Published:** 2026-10-16

**Authors:** Zhenxiao Wang, Mo Li, Zhenyu Wang, Ying Wang

**Affiliations:** 1 Department of Hepatobiliary and Pancreatic Surgery, The Second Hospital of Jilin University, Changchun, Jilin, China; 2 Department of Thyroid Surgery, The Second Hospital of Jilin University, Changchun, Jilin, China; 3 Department of Breast Surgery, The Second Hospital of Jilin University, Changchun, Jilin, China

**Keywords:** immunosuppression, immunotherapy, metabolic reprogramming, nanomedicine, polymer-based drug delivery systems, triple-negative breast cancer, tumor microenvironment

There was a mistake in **Figure 3** as published. During the manuscript revision process, we accidentally uploaded the same image for both **Figure 3** andFigure 4. The corrected **Figure 3** appears below.

**Figure 3:**
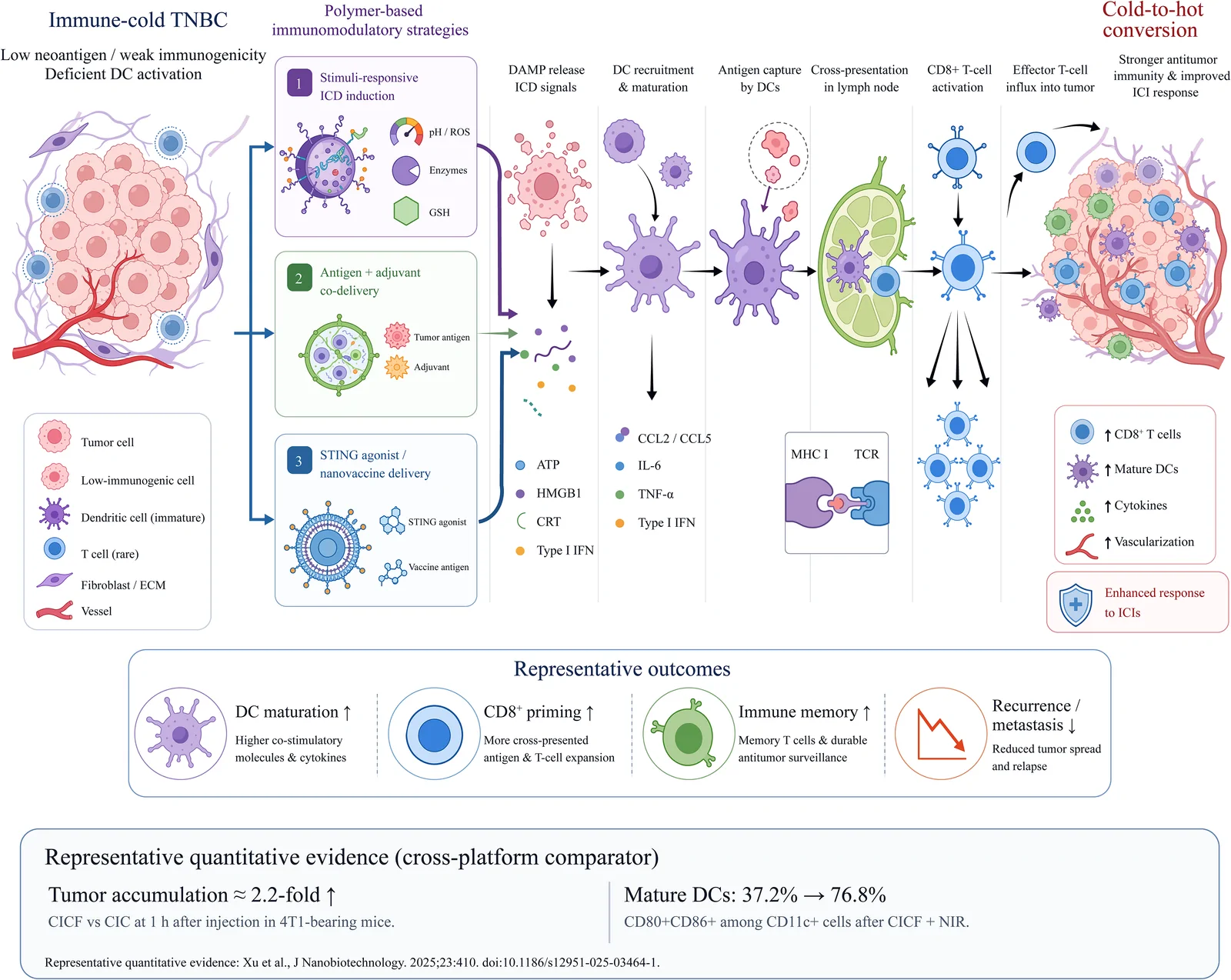
Restoring immunogenicity and antigen presentation through barrier-matched polymer design. Stimuli-responsive ICD, antigen/adjuvant co-delivery and STING-oriented delivery reinforce complementary steps of the cancer-immunity cycle, including DAMP release, dendritic-cell recruitment and maturation, antigen capture and cross-presentation, CD8+ T-cell priming and effector-cell influx. The overall design goal is conversion of immune-cold TNBC into a more immune-active state with improved responsiveness to immune checkpoint blockade. A representative recent cross-platform study is included as a quantitative comparator, showing approximately 2.2-fold higher tumoral fluorescence at 1 h and an increase in mature DCs from 37.2% to 76.8%. Data summarized from Xu et al., J Nanobiotechnology. 2025;23:410.

The original version of this article has been updated.

